# Niche Partitioning of the N Cycling Microbial Community of an Offshore Oxygen Deficient Zone

**DOI:** 10.3389/fmicb.2017.02384

**Published:** 2017-12-05

**Authors:** Clara A. Fuchsman, Allan H. Devol, Jaclyn K. Saunders, Cedar McKay, Gabrielle Rocap

**Affiliations:** School of Oceanography, University of Washington, Seattle, WA, United States

**Keywords:** denitrification, anammox, Eastern Tropical North Pacific, oxygen minimum zone, particles

## Abstract

Microbial communities in marine oxygen deficient zones (ODZs) are responsible for up to half of marine N loss through conversion of nutrients to N_2_O and N_2_. This N loss is accomplished by a consortium of diverse microbes, many of which remain uncultured. Here, we characterize genes for all steps in the anoxic N cycle in metagenomes from the water column and >30 μm particles from the Eastern Tropical North Pacific (ETNP) ODZ. We use an approach that allows for both phylogenetic identification and semi-quantitative assessment of gene abundances from individual organisms, and place these results in context of chemical measurements and rate data from the same location. Denitrification genes were enriched in >30 μm particles, even in the oxycline, while anammox bacteria were not abundant on particles. Many steps in denitrification were encoded by multiple phylotypes with different distributions. Notably three N_2_O reductases (*nosZ*), each with no cultured relative, inhabited distinct niches; one was free-living, one dominant on particles and one had a C terminal extension found in autotrophic S-oxidizing bacteria. At some depths >30% of the community possessed nitrite reductase *nirK*. A *nirK* OTU linked to SAR11 explained much of this abundance. The only bacterial gene found for NO reduction to N_2_O in the ODZ was a form of *qnorB* related to the previously postulated “nitric oxide dismutase,” hypothesized to produce N_2_ directly while oxidizing methane. However, similar *qnorB-like* genes are also found in the published genomes of many bacteria that do not oxidize methane, and here the *qnorB-like* genes did not correlate with the presence of methane oxidation genes. Correlations with N_2_O concentrations indicate that these *qnorB-like* genes likely facilitate NO reduction to N_2_O in the ODZ. In the oxycline, *qnorB-like* genes were not detected in the water column, and estimated N_2_O production rates from ammonia oxidation were insufficient to support the observed oxycline N_2_O maximum. However, both *qnorB-like* and *nosZ* genes were present within particles in the oxycline, suggesting a particulate source of N_2_O and N_2_. Together, our analyses provide a holistic view of the diverse players in the low oxygen nitrogen cycle.

## Introduction

Naturally occurring oxygen deficient zones (ODZs), defined here as water containing <10 nM oxygen, constitute <1% of the ocean volume, but contribute 30–50% of N loss from the marine system through N_2_ production (DeVries et al., 2013). In the absence of oxygen, microbes in these waters use a variety of different terminal electron acceptors, including oxidized nitrogen (nitrate and nitrite), resulting in the production of both N_2_ and the greenhouse gas N_2_O. Due to the temperature-dependent solubility of oxygen, concentrations are predicted to be further reduced in the future, increasing the size of ODZs (Deutsch et al., 2011) with implications for increased N_2_ production and consequent N limitation of global primary production. Thus, understanding the microbial community responsible for N_2_ production is important to predicting the nitrogen budget of a changing ocean.

Denitrification and anammox are the two known pathways for N_2_ production. Denitrification is the multi-step reduction of ${\text{NO}}_{3}^{-}$ to N_2_ paired with oxidization of organic matter, reduced sulfur, or methane. Nitrate is reduced through four reduction steps, each encoded by a different enzyme. However, not all denitrifying organisms possess all the enzymes needed to reduce ${\text{NO}}_{3}^{-}$ all the way to N_2_. Many encode genes for only a subset of the steps and rely on other microbes for the production or consumption of their reactants and products (Zumft, 1997). This separation of individual reactions may be possible because most steps in denitrification occur in the periplasm (Zumft, 1997), which should ease transport of reactants and products across the outer membrane. Genes in the denitrification pathway are found widespread throughout the microbial tree of life (Zumft, 1997). Some horizontal gene transfer of denitrification genes has occurred and some denitrification genes have even been found on plasmids (Zumft, 1997). However, duplication and divergence of genes followed by incomplete lineage sorting maybe more important than horizontal gene transfer in accounting for the diversity of denitrification genes (Jones et al., 2008). Denitrifiers are also metabolically diverse, with heterotrophic denitrifiers coupling N reduction with organic matter oxidation and autotrophic denitrifiers coupling N reduction with sulfur or methane oxidation to fuel carbon fixation (Hannig et al., 2007; Ettwig et al., 2010; Babbin et al., 2014). In contrast, autotrophic bacteria in the order Brocadiales in the Planctomycetes phylum are the other organisms known to carry out the anammox process, which involves reduction of ${\text{NO}}_{2}^{-}$ with ${\text{NH}}_{4}^{+}$ to produce N_2_. The process occurs in an internal compartment where the membrane is composed of ladderane lipids; these tightly fitting lipids are needed to isolate the toxic intermediate hydrazine (N_2_H_4_) from the rest of the cell and prevent hydrazine leakage, which would make anammox energetically unfeasible (Sinninghe Damsté et al., 2002). This complex cellular structure may be one reason why anammox is restricted phylogenetically, with *Scalindua* the only genera of anammox bacteria thus far found in the marine environment (van de Vossenberg et al., 2013).

The relative importance of anammox compared to denitrification in marine N_2_ production is still unclear (Lam et al., 2009; Ward et al., 2009; Dalsgaard et al., 2012). If the breakdown of organic matter by heterotrophic denitrifiers is the source of ammonia to anammox, then stoichiometrically heterotrophic denitrification should contribute 70% and anammox 30% to N_2_ production (Devol, 2003) though the consumption of nitrite through nitrite oxidation can shift this ratio to >40% anammox (Penn et al., 2016). However, in older studies, denitrifiers, identified molecularly by targetting the nitrite reductase gene *nirS* were in low abundance (Lam et al., 2009, 2011; Jensen et al., 2011; Jayakumar et al., 2013; Kalvelage et al., 2013) and had low diversity (Bowen et al., 2015). More recently, using primer-independent approaches the copper-dependent nitrite reductase encoded by *nirK* was found in addition to *nirS*, and was more abundant (Ganesh et al., 2014, 2015; Glass et al., 2015; Lüke et al., 2016). In the Arabian Sea and ETSP, this *nirK* was predominantly from ammonia oxidizing archaea while in the ETNP *nirK* from nitrite oxidizer *Nitrospina* was found in addition (Glass et al., 2015; Lüke et al., 2016). However, neither ammonia oxidizing archaea or nitrite oxidizing bacteria are complete denitrifiers, so the role of *nirK* in N_2_ production is still unclear.

Anammox and denitrifying bacteria may have different niches in the ODZ. Size fractionated studies in the ETSP and the Black Sea indicate that marine anammox bacteria are primarily free-living (Fuchsman et al., 2012b; Ganesh et al., 2014, 2015) as are nitrate reducers (Ganesh et al., 2015), but the last two steps in the denitrification pathway are enriched in >1.6 μm suspended particles (Ganesh et al., 2014, 2015). The most abundant nitrate reducer in the ETNP was a free-living SAR11 in the ODZ which has two different nitrate reductases transferred from quite distinct bacteria (gammaproteobacteria and candidate phyla OP1) but lacks genes for the last two steps in denitrification (Tsementzi et al., 2016). The organisms containing genes for the last step in denitrification, N_2_O reductase *nosZ*, are largely unknown (Ganesh et al., 2014, 2015). The addition of sterilized sediment trap material significantly increased denitrification and anammox rates in all three marine ODZs (Babbin et al., 2014; Chang et al., 2014). Denitrification rates increase more than anammox (Babbin et al., 2014; Chang et al., 2014), probably because denitrification uses organic matter directly while anammox uses ammonia from organic matter degradation. N_2_ production has also been found inside particles composed of diatom aggregates or zooplankton carcasses at hypoxic oxygen concentrations (Stief et al., 2016, 2017).

Denitrification in marine ODZs is generally attributed to heterotrophic denitrification. However, some autotrophic denitrification has been measured in incubations with sulfide in the coastal ETSP (Canfield et al., 2010) and autotrophic N_2_ production by methane oxidizers has been proposed in the ETNP (Padilla et al., 2016). *Cand*. Methylomirabilis oxyfera, isolated from fresh water, can oxidize methane with nitrite, forming N_2_ from nitrite without an N_2_O intermediate (Ettwig et al., 2010). The gene in question was dubbed nitric oxide dismutase (*nod*) based on *in silico* analysis (Ettwig et al., 2012) and has been found in both transcript and gene data from the ETNP (Padilla et al., 2016). However, rates of methane oxidation in the ETNP ODZ are the slowest rates measured in the ocean (0.034–15 × 10^−3^ nmol CH_4_ L^−1^ d^−1^), suggesting that methane oxidation is not a dominant process in the ETNP (Pack et al., 2015).

Nitrite and ammonium oxidation have been shown previously in the upper ODZ in the ETNP (Peng et al., 2015; Garcia-Robledo et al., 2017). Although the ETNP ODZ contains <10 nM oxygen (Tiano et al., 2014), the low oxygen K_m_ for nitrite oxidation 0.5 ± 4 nM (Bristow et al., 2016), indicates nitrite oxidation is possible when oxygen concentrations below detection. Oxygen needed for nitrite oxidation may be provided by photosynthesis by *Prochlorococcus* in the upper ODZ or by mixing of waters from the oxycline above (Peters et al., 2016; Garcia-Robledo et al., 2017). The K_m_ for ammonia oxidizers is 333 ± 130 nM, which is significantly higher than for nitrite oxidation (Bristow et al., 2016). Correspondingly, ammonia oxidation rates drop off more rapidly than nitrite oxidation rates in the upper ODZ (Peng et al., 2015).

Here, we take a holistic approach to the low oxygen nitrogen cycle using metagenomics combined with existing chemical and rate measurements. We performed a phylogenetic analysis of key functional genes in the N cycle from assemblies of a metagenomic 10-depth profile in the water column and the >30 μm particle attached community at three key depths in the offshore ETNP ODZ. We identified previously unknown phylotypes for many key N cycling genes. We then used a read placement approach to quantify the distributions of all phylotypes for each gene in a semi-quantitative manner and found multiple differences both with respect to depth and presence in the particle attached vs. whole water community.

## Methods

### Hydrographic data

Samples were collected in April 2012 aboard the R/V Thompson TN278 using 10 L Niskin bottles on a 24 bottle CTD-rosette. A Seabird 911 Conductivity Temperature Density meter, a Seabird SBE 43 Dissolved Oxygen Sensor, a WETLabs ECO Chlorophyll Fluorometer, and a Biospherical/Licor PAR/Irradiance Sensor were attached to the rosette. Nutrient samples were filtered (GF/F glass fiber; nominal pore size 0.7 μm) before analysis. Nutrient analyses were performed by members of the University of Washington Marine Chemistry Laboratory on board the ship using a Technicon AAII system as described by the World Ocean Circulation Experiment (WOCE) Hydrographic Program protocol (Gordon et al., 1995). Ammonium was measured on board the ship using the fluorometric orthophthaldialdehyde (OPA) method due to the low detection limit (10 nM) of this method (Holmes et al., 1999). Hydrographic and nutrient data from the cruise are deposited at http://data.nodc.noaa.gov/accession/0109846. Eight day averaged satellite chlorophyll (April 7, 2012) from satellite MODIS Aqua R2014 was downloaded from http://www.science.oregonstate.edu/ocean.productivity.

### N_2_ gas concentrations

N_2_ gas samples were collected from St 136 and analyzed as in Chang et al. (2010, 2012). Very briefly, duplicate gas samples were collected in evacuated 185 mL glass flasks sealed with a Louwers-Hapert valve and containing dried mercuric chloride as a preservative. To prevent air contamination when sampling, samples were transferred from the Niskin bottle to the sample flask under a local CO_2_ atmosphere. Head-space gases were cryogenically processed to completely remove CO_2_ and residual water vapor and run through an inline CuO furnace to remove oxygen. Then gases were measured at the Stable Isotope Lab, School of Oceanography, University of Washington on a Finnigan Delta XL isotope ratio mass spectrometer. The anoxic samples were measured against a standard containing zero oxygen. Background N_2_/Ar ratios from representative water outside the ODZ were removed as in Chang et al. (2012), leaving concentrations of biologically produced N_2_.

### Metagenomic data

DNA samples were obtained from station 136 (106.543° W 17.043° N; cast 136) at 10 depths including the oxycline and anoxic zones. Two liters of Niskin water were vacuum filtered onto a 0.2 μm SUPOR filter. At station BB2, a nearby station (107.148° W 16.527° N; cast 141), ~4 L were prefiltered through >30 μm filters at 100, 120, and 150 m depths and subsequently filtered onto 0.2 μm SUPOR filters. >30 μm filters were sequenced for all three depths, but only the <30 μm filter from 120 m was sequenced. Particles >30 μm should be composed of sinking as well as large suspended particles (Clegg and Whitfield, 1990). Station 136 and BB2 were only 83 km apart and hydrographic conditions were very similar (Figures S1, S2). DNA was extracted from filters using freeze thaw followed by incubation with lysozyme and proteinase K and phenol/chloroform extraction. A Rubicon THRUPLEX kit was used for library prep using 50 ng of DNA per sample. Four libraries were sequenced on an Illumina HiSeq 2500 in rapid mode (~25 million 150 bp paired-end reads per sample) at Michigan State. The other 10 libraries were sequenced on an Illumina HiSeq 2500 in high output mode (~40–70 million 125 bp paired-end reads per sample) at the University of Utah (Table S1). Sequences were quality checked, trimmed, and remaining adapter sequences were removed using Trimmomatic (Bolger et al., 2014). Paired reads that overlapped were combined with Flash (Magoc and Salzberg, 2011).

Metagenomic sequences from each sample were assembled independently into larger contigs. For de novo assembly we pre-processed reads with the khmer software package (Crusoe et al., 2015), first using normalize-by-median which implements a Digital normalization algorithm (Brown et al., 2012) to reduce high coverage reads to 20x coverage, followed by filter-abund.py to trim reads of kmers with an abundance below 2, and finally we used filter-below-abund.py to trim kmers with counts above 50 (Zhang et al., 2015). We assembled the khmer processed reads with the VELVET (1.2.10) assembler (Zerbino, 2010), using a kmer size of 45. The N50, or median length, for assembled contigs ranged between 1,300 and 1,800 bp in the anoxic zone, with ~30% of reads assembled (Table S1). The Prokka annotation pipeline (Seemann, 2014) was used for gene calling, which relies on the Prodigal algorithm for identification of coding sequence coordinates on the contigs (Hyatt et al., 2010), and preliminary functional annotation identified through similarity searching with BLAST (Altschul et al., 1997) against UniProt (Apweiler et al., 2004) and RefSeq (Pruitt et al., 2007) databases and with HMMER v. 3.1 (Eddy, 2011) against protein domain databases Pfam (Punta et al., 2012) and TIGRFAMs (Haft et al., 2013). ETNP 2012 metagenomic reads and assembled contigs can be found at NCBI GenBank bioproject PRJNA350692.

For each gene of interest, a maximum likelihood amino acid phylogenetic tree was constructed using published full-length gene sequences as well as full or nearly full-length sequences assembled from the metagenomes themselves. Rather than rely on Prokka annotations, potential gene sequences of interest were identified from the metagenome assemblies by searching a custom blast database (Altschul et al., 1997) of all our assembled open reading frames as called by Prodigal, using representative published sequences from each section of the phylogenetic tree as query sequences. All sequences with an e-value cut-off of < −60 were included in the phylogenetic tree for further identification. Assembled genes with Ns were removed. In addition, full-length published gene sequences of closely related genes to the gene of interest were included to act as outgroups in the trees. All assembled sequences recruited from blast were combined with the previously published full-length gene sequences and aligned in amino acid space with MUSCLE v. 3.8.1551 (Edgar, 2004). Maximum likelihood phylogenetic trees were constructed with the reference sequence alignments of the genes of interest using the program RAxML v. 8.1.20 (Stamatakis, 2014). In this process, sequences with exactly identical amino acid sequences were de-duplicated (Stamatakis, 2014). The trees were constructed with a gamma model of rate heterogeneity, and appropriate amino acid substitution models were determined for each tree, and bootstrap analyses (*n* = 100) were performed.

A phylogenetic placement approach was used to characterize short metagenomic reads related to the targeted genes of interest (Berger et al., 2011) in a semi-quantitative and phylogenetically specific manner (Saunders and Rocap, 2016). For read placement, the short metagenomic reads were recruited via tblastn search of the metagenomes using an e-value cut-off of <-5 (Altschul et al., 1997). The recruited reads were trimmed to the edge of the gene of interest to remove any overhang of up or down-stream sequence, trimmed to the proper reading frame of the blast results, and converted to amino acid space. Any sequence ambiguities and stop codons were removed. Presence of sequence ambiguities and stop codons were negligible. Only sequences longer than 100 bp (33 amino acids) after quality trimming were used for placement analysis. These amino acid translated reads were aligned to the reference sequences in amino acid space using PaPaRa: Parsimony-based Phylogeny-Aware Read Alignment program v. 2.5 (Berger and Stamatakis, 2011). Following the PaPaRa alignment, paired end reads were combined into one sequence in the same alignment using a python script and placed as one read on the tree using the EPA: Evolutionary Placement Algorithm portion of RAxML (Stamatakis, 2014). Reads that placed with outgroups on each phylogenetic tree were not counted toward that gene's total. This was particularly important for closely related genes such as nitrate reductase *narG*, with the *nxrA* outgroup and for nitrite oxidoreductase *nxrB* with the *narH* outgroup.

To take into account differences in sequencing effort between samples and recruitment capacity among the different genes, read placement length normalized occurrence of the target genes were normalized to the length normalized abundance of the universal single copy core gene RNA polymerase (*rpoB;* Figure S3).

$$\begin{array}{l} \%\;prokaryotic\;community=\frac{\frac{Gene\;A\;reads}{Length\;A}}{\frac{rpoB\;reads}{Length\;rpoB}}. \end{array}$$

Since RNA polymerase is, to our knowledge, present as a single copy gene in all bacterial and archaeal genomes, normalization of a target gene occurrence to RNA polymerase abundance indicates what percentage of the prokaryotic community contains the gene of interest. This normalization allows our placements to be quantitative in a relative manner. However, this analysis does not take into account that the density of prokaryotic cells may, and undoubtedly do, change with depth.

Several papers have been published from this cruise, and we compare our metagenomic data to previous rate, qPCR, and lipid data. For clarification, these data and their references are listed in Table S2.

### Previously published transcripts

In order to assess whether phylotypes observed in our metagenomes were expressed in the environment, we applied the same read placement approach to previously published metatranscriptomic data. Transcripts from 2 size fractions (0.2–1.6 and 1.6–30 μm) at five low oxygen depths from a coastal station in the ETNP in 2013 (18° 54.0′ N, 104° 54.0′ W) (Ganesh et al., 2015) were placed on our phylogenetic trees using methods as above. Transcript libraries had ~120,000 reads per sample.

## Results and discussion

**Figure 1:**
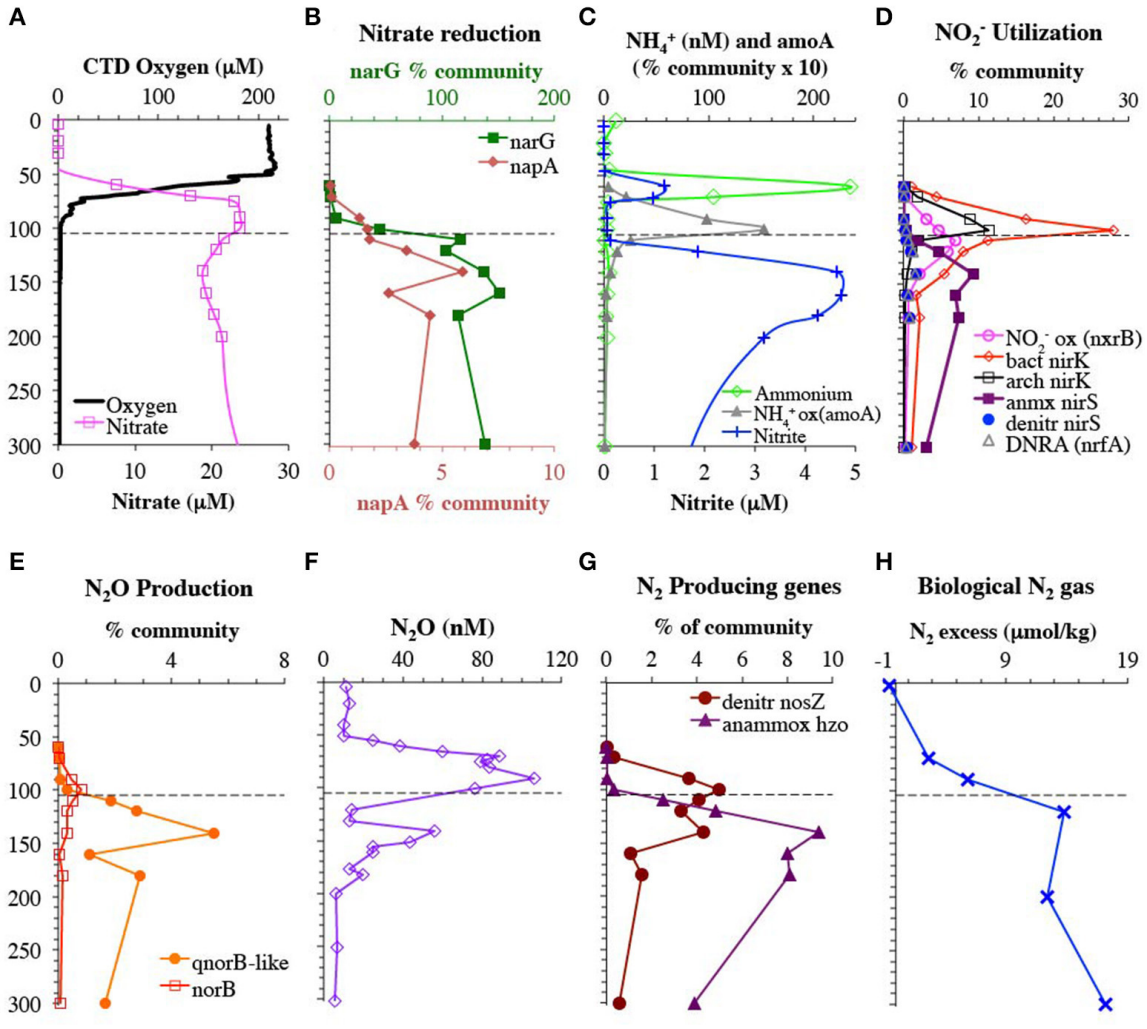
Depth profiles of metagenomic reads encoding for enzymes used in the low oxygen N cycle and relevant chemical species. **(A)** CTD oxygen and nitrate concentrations from St 136. **(B)** Genes used in nitrate reduction. Genes for nitrate reductases *narG* and *napA* are shown on different axes for clarity. **(C)** Nitrite and OPA ammonium concentrations from St 136 as well as the gene for ammonia monooxygenase (*amoA*) used in ammonia oxidation. **(D)** Genes used for nitrite utilization: nitrite reduction to NO (bacterial and archaea *nirK*, anammox *nirS*, and bacterial *nirS*), nitrite reduction to ammonium (DNRA: *nrfA*) and nitrite oxidation (*nxrB*). **(E)** N_2_O producing genes: NO reductases *norB* and *qnorB*-like. **(F)** N_2_O concentration profile from St BB2 (Peng et al., 2015). **(G)** N_2_ producing genes: N_2_O reductase (*nosZ*) represents denitrification and hydrazine oxidoreductase (*hzo*) represents anammox. **(H)** Biological N_2_ gas from St 136. Dashed line represents the top of the ODZ, which is at 105 m (Tiano et al., 2014). % Community is calculated in comparison to the single copy core gene RNA polymerase (*rpoB*).

We occupied 2 stations in the offshore Eastern Tropical North Pacific in April 2012. At these stations, the depth of anoxia was ~105 m according to oxygen measurements with a STOX sensor (detection limit 2 ± 5 nM O_2_; Tiano et al., 2014). The oxycline, where oxygen concentrations decrease rapidly, extended from 60 to 100 m (Figure 1A). STOX oxygen concentrations were 4.7 and 0.8 μM at 90 and 100 m in the lower oxycline (Tiano et al., 2014). Ammonium concentrations, as determined by the extremely sensitive OPA method, were undetectable in the anoxic zone (Figure 1C), but the nitrite maximum reached nearly 5 μM at 150 m (Figure 1C). N_2_O concentrations had the usual large maximum in the oxycline, but also had a second smaller maximum at 140–150 m in the anoxic zone (Figure 1F; Peng et al., 2015). Biological N_2_ gas increased in the anoxic zone and was between 10 and 11 μM (Figure 1H). To characterize the functional and taxonomic diversity of this oligotrophic ODZ community, we constructed and sequenced metagenomes from whole water from 10 depths (60–300 m) at station 136 and from >30 μm particles collected from Niskin bottles at 3 depths at station BB2, which was close to and very similar physiochemically to station 136 (Figure S2). We classified short read sequences by both function and phylotype by placing reads on reference trees constructed from full length genes, including those assembled from our metagenomes.

### Community structure of free living and particle communities

**Figure 2:**
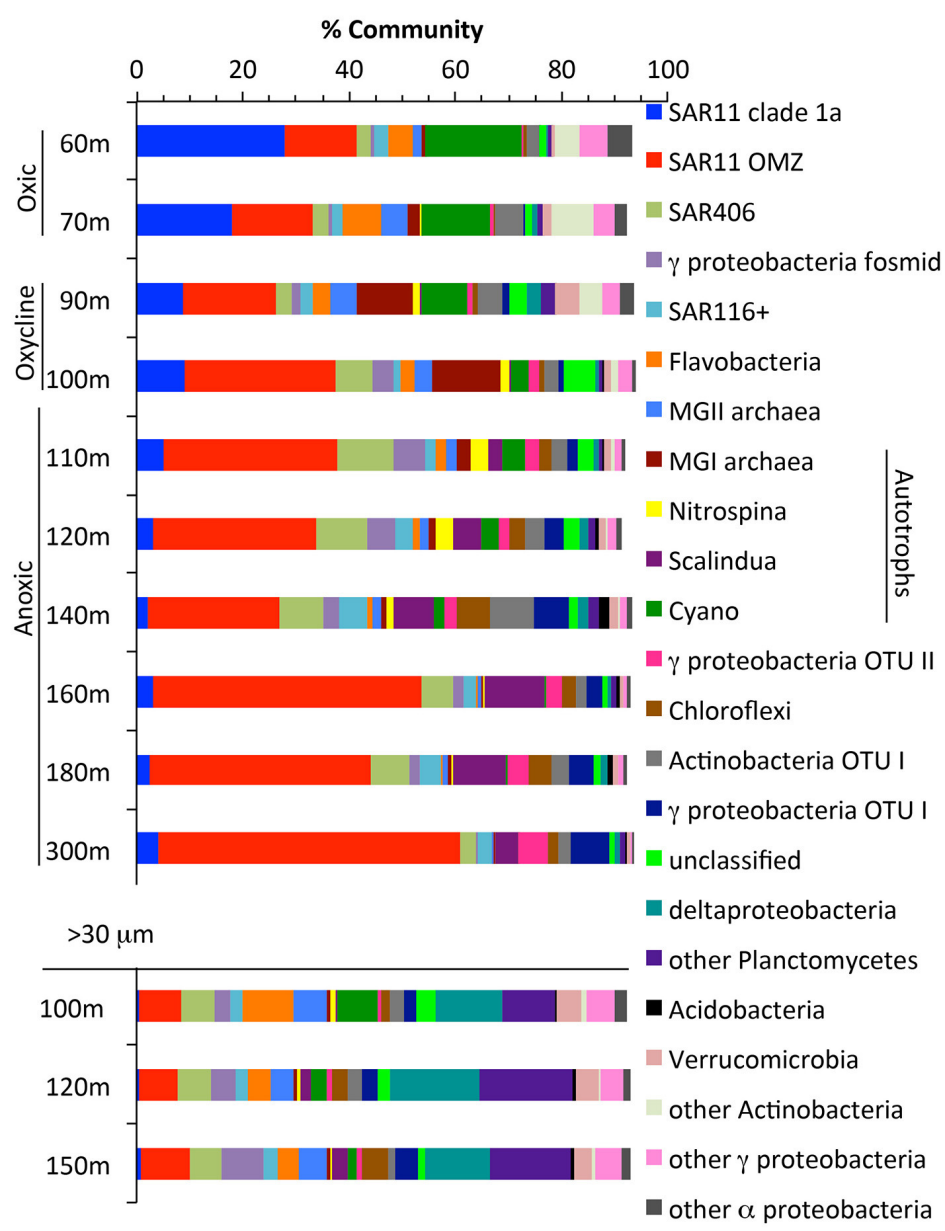
The community in the water column and in >30 micron particles as determined by RNA polymerase gene *rpoB*. Groups found on the *rpoB* phylogenetic tree (Figure S2). Known autotrophs are labeled in the legend. Gamma fosmid represents relatives of HOT fosmid GU567967, a heterotrophic Salinisphaeraceae.

We examined community structure using the RNA polymerase (*rpoB*) gene, found in all archaeal and bacterial genomes (Figure 2, Figure S3). SAR11 was the most abundant clade overall, making up 60% of the community at 300 m. SAR11 has previously been found to be 10–40% of the community in ODZs (Tsementzi et al., 2016). Other notable heterotrophic clades present included SAR406, SAR116, Marine Group II euryarchaeota, and Flavobacteria (Figure 2). Autotrophic microbes present included Cyanobacteria (photosynthesis), Marine Group I Thaumarchaeota (ammonia oxidation), *Nitrospina* (nitrite oxidation), and *Cand*. Scalindua (anammox), and together they made up <20% of the community in the oxycline and anoxic water column (Figure 2). Autotrophic S oxidizers are also known to be active in ODZs (Stewart et al., 2012), but known S oxidizers, including SUP05, were not identified here. However, it should be noted that a large number of the *rpoB* sequences were novel phylotypes including novel clades of Actinobacteria, Chloroflexi, Acidobacteria, and gammaproteobacteria whose metabolic lifestyles are unknown (Figure S3).

Particles may be hotspots of heterotrophic activity in the ocean and harbor a microbial community distinct from those free-living in the water column (Delong et al., 1993; Ploug et al., 1999). To further examine the microbial community on particles that make up the sinking organic matter in the ODZ, we sequenced metagenomes from >30 μm particles collected from Niskin bottles at 3 depths at the offshore station BB2. Particles >30 μm should be composed of sinking as well as large suspended particles (Clegg and Whitfield, 1990). Estimates of 16S rRNA abundance in the ETNP using qPCR indicated that 8–15 times more 16S rRNA was found per mL in free-living (<1.6 μm) communities compared to particle (>1.6 μm) fractions (Ganesh et al., 2015). Thus, the free-living community likely dominates bulk water samples. In our dataset, the microbial community on particles (>30 μm) differed from the community in the bulk water (Figure 2). Among others, SAR11, Marine Group I archaea (ammonia oxidizing) and *Cand*. Scalindua (anammox), all known to be free-living (Fuchsman et al., 2011, 2012b, Ganesh et al., 2014, 2015), were a much smaller proportion of the particle community. Delta proteobacteria, Planctomycetes, Flavobacteria, Verrucomicrobia, and MGII euryarchaeota were enriched in particles, consistent with observations in other environments (Delong et al., 1993; Fuchsman et al., 2011, 2012b; Ganesh et al., 2014; Glass et al., 2015; Orsi et al., 2015). In contrast to previous observations in the ETNP (Ganesh et al., 2015), the nitrite-oxidizer *Nitrospina* was not enriched in particles. This could be due to the different size filter used to define “particles” (1.6 vs. 30 μm used here) because individual *Nitrospina* cells can be 6 μm long (Spieck and Bock, 2015).

### Genetic capacity for denitrification enhanced on particles

**Figure 3:**
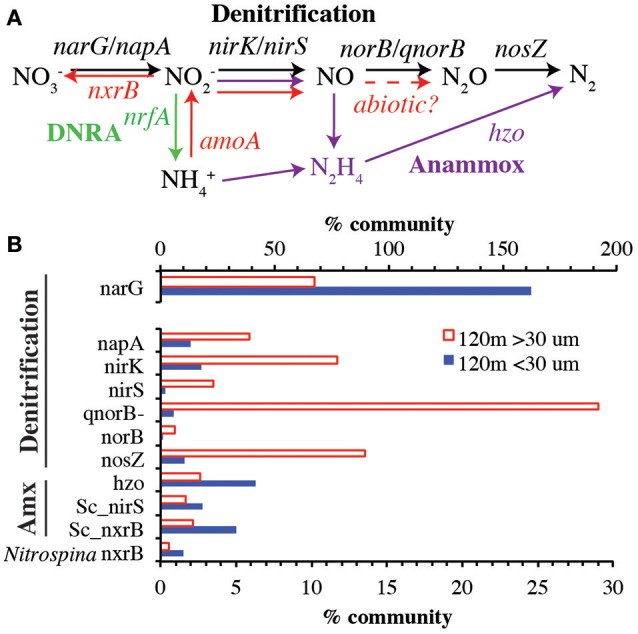
**(A)** The low oxygen N cycle with genes examined in this paper indicated. Black arrows indicate denitrification. Purple arrows indicate anammox. Red arrows indicate processes mediated by ammonia and nitrite oxidizing organisms. **(B)** Comparison of genes between >30 μm and <30 μm fractions at 120 m. Note that nitrate reduction gene *narG* is on its own axis. *amoA* and *nrfA* were <1% of community at 120 m, so are not shown. Sc_*nirS* and Sc_*nxrB* indicate genes belonging to anammox bacteria *Cand*. Scalindua. *Nitrospina nxrB* represents nitrite oxidizing bacteria. % Community is calculated in comparison to the single copy core gene RNA polymerase (*rpoB*).

To examine the role of sinking and large suspended particles in production of N_2_ by the ODZ microbial community, we mapped metagenomic reads onto reference phylogenetic trees constructed for key genes in the anoxic N cycle (Figure 3). A comparison of >30 μm and <30 μm communities at 120 m revealed that most of the genes encoding the steps of denitrification were enriched in the particulate fraction while anammox genes and the nitrate reductase *narG* represented a greater fraction of the community in the free-living fraction (Figure 3). The particle associated denitrification genes were not present in equal amounts, ranging from more than 25% of the community possessing the NO reduction enzyme (*qnorB-like*) to <5% with the nitrite reductase *nirS*. Overall, nitrate reductase *narG* was the most abundant gene examined, present in the equivalent of >150% of the free-living community and >50% of the particle community. This apparent overestimate is due to the presence of multiple copies of *narG* in many microbial genomes, including the abundant ODZ SAR11 phylotypes (Tsementzi et al., 2016), but nevertheless underscores the important role of nitrate reduction in this environment, both on particles and in the water column. The habitat partitioning observed here, with anammox and *narG* primarily in the free living fraction and other denitrification genes enriched in sinking particles is consistent with data from >1.6 μm suspended particles (Ganesh et al., 2014, 2015). In the coastal ETNP, removal of >1.6 μm particles decimated nitrate reduction rates (Ganesh et al., 2015), reinforcing the idea that even the free-living bacteria in the ODZ are dependent on organic matter fluxes. Consistent with the *rpoB* data, nitrite oxidoreductase (*nxrB*) for nitrite-oxidizer *Nitrospina* was more abundant in the <30 μm fraction.

### Gene depth profiles

We next examined the depth distribution and taxonomic diversity of these same key genes (Figure 3A) in the oxycline and upper 200 m of the ODZ water column. We compare these depth profiles to measurements of chemical species that serve as reactants and products for the reactions catalyzed by the enzymes these genes encode (Figure 1).

#### Nitrification genes

The ammonia monooxygenase (*amoA*) gene for ammonia-oxidizing archaea had a maximum of 15% of community at 100 m at the bottom of the oxycline (Figure 1C). This maximum in *amoA* was below the primary maxima in ammonium and nitrite concentrations found at 60 m. This depth profile combined with ammonia oxidation rates from Peng et al. (2015) indicate that nitrite is being produced in the 80–100 m region, despite the lack of measurable nitrite in this region. The gene encoding the nitrite oxidizing enzyme nitrite oxidoreductase (*nxrB*) is unmeasurable at 70 m, but 3% of the community at 90 m, and had a maximum of 7% of the community in the ODZ at 110 m that can be solely attributed to *Nitrospina* (Figure 1D, Figure S6). The presence of *Nitrospina nxrB* in the upper ODZ is consistent with *nxrB* transcript data from the ETNP (Garcia-Robledo et al., 2017). The presence of *Nitrospina* may help explain the lack of measurable nitrite in the 80–100 m region.

#### Nitrate reduction

**Figure 4:**
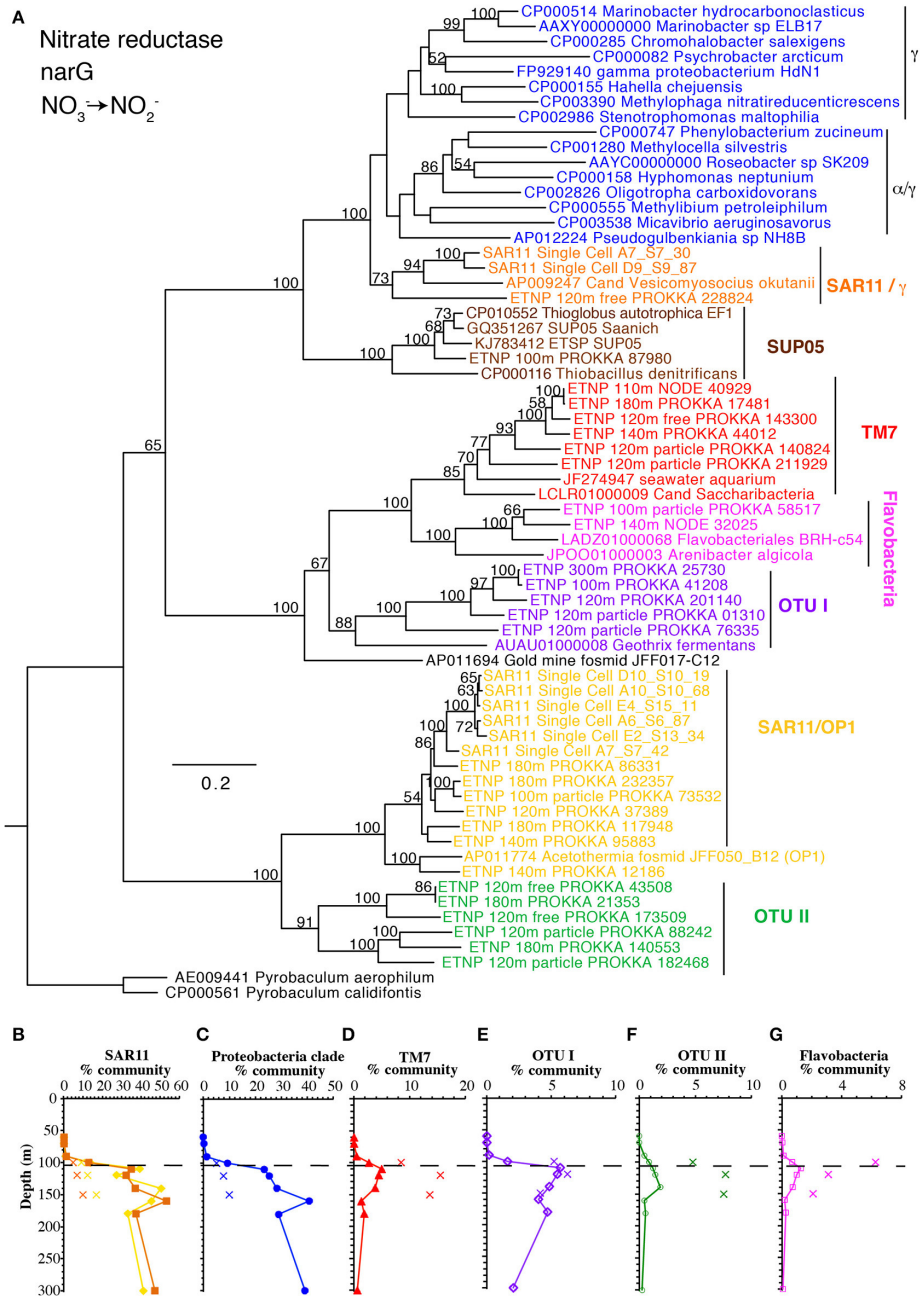
**(A)** Phylogenetic tree of membrane bound nitrate reductase gene *narG*. Names with ETNP indicate sequences assembled from our metagenomic samples. Labels and colors on the tree match phylotype depth profiles **(B–G)**. **(B)** Depth profile of both forms of *narG* belonging to SAR11. **(C)** Combined profile of α and γ proteobacteria, **(D)** uncultured group TM7, **(E)** unknown OTU I, **(F)** unknown OTU II, and **(G)** Flavobacteria. In all depth profiles, Xs indicate particulate (>30 μm) samples. Dashed line indicated the top of the ODZ. % Community is calculated in comparison to the single copy core gene RNA polymerase (*rpoB*).

The first step in denitrification, nitrate reduction to nitrite, can be carried out by nitrate reductases encoded by either *narG* or *napA*, both of which were detected here. However, *narG* was an order of magnitude more abundant than all the other denitrification genes (Figure 1B). The *narG* maximum at 160 m corresponded with a reduction in nitrate concentrations and the secondary nitrite maximum (Figures 1A–C), implying nitrate reduction activity. Again, after normalizing with the single copy core gene, more than 100% of the community contained the *narG*, implying multiple copies per genome in some bacteria. SAR11, which is very abundant in our *rpoB* data (Figure 2), is known to have two distinct *narG* in the same cell (Tsementzi et al., 2016) and we found both SAR11 *narG* types here along with six other phylotypes (Figure 4). If we assume that all SAR11 genomes have 2 types of *narG*, we calculate that 75–105% of the total microbial community has *narG*. This number may suggest that other groups in addition to SAR11 also possess duplicate copies of *narG*. In contrast, the two phylotypes of *napA* totaled only 5% of the community in the ODZ (Figure 1B, Figure S4). The capacity for nitrate reduction is clearly prevalent in the community. Examination of selected long contigs containing *narG* indicated multiple nitrate reductase subunits and at least one nitrate transporter (*narU, narT*, or *narK*) in all cases (Figure S5). Contigs associated with OTU I contained a transposase, indicating the potential for horizontal gene transfer (Figure S5).

#### Nitrite reduction

The nitrite produced by nitrate reduction can have many fates, including further reduction to NO, oxidation back to nitrate, and reduction to ammonia. We found evidence for existence of all of these pathways, in differing amounts. As mentioned previously, the gene encoding the nitrite oxidizing enzyme nitrite oxidoreductase (*nxrB*) had a maximum in the upper ODZ (Figure 1D). In contrast *nrfA*, encoding the DNRA nitrite reductase to ammonia, was present but always <2% of the community (Figure 1D). Only one phylotype of *nrfA* was present (Figure S7).

**Figure 5:**
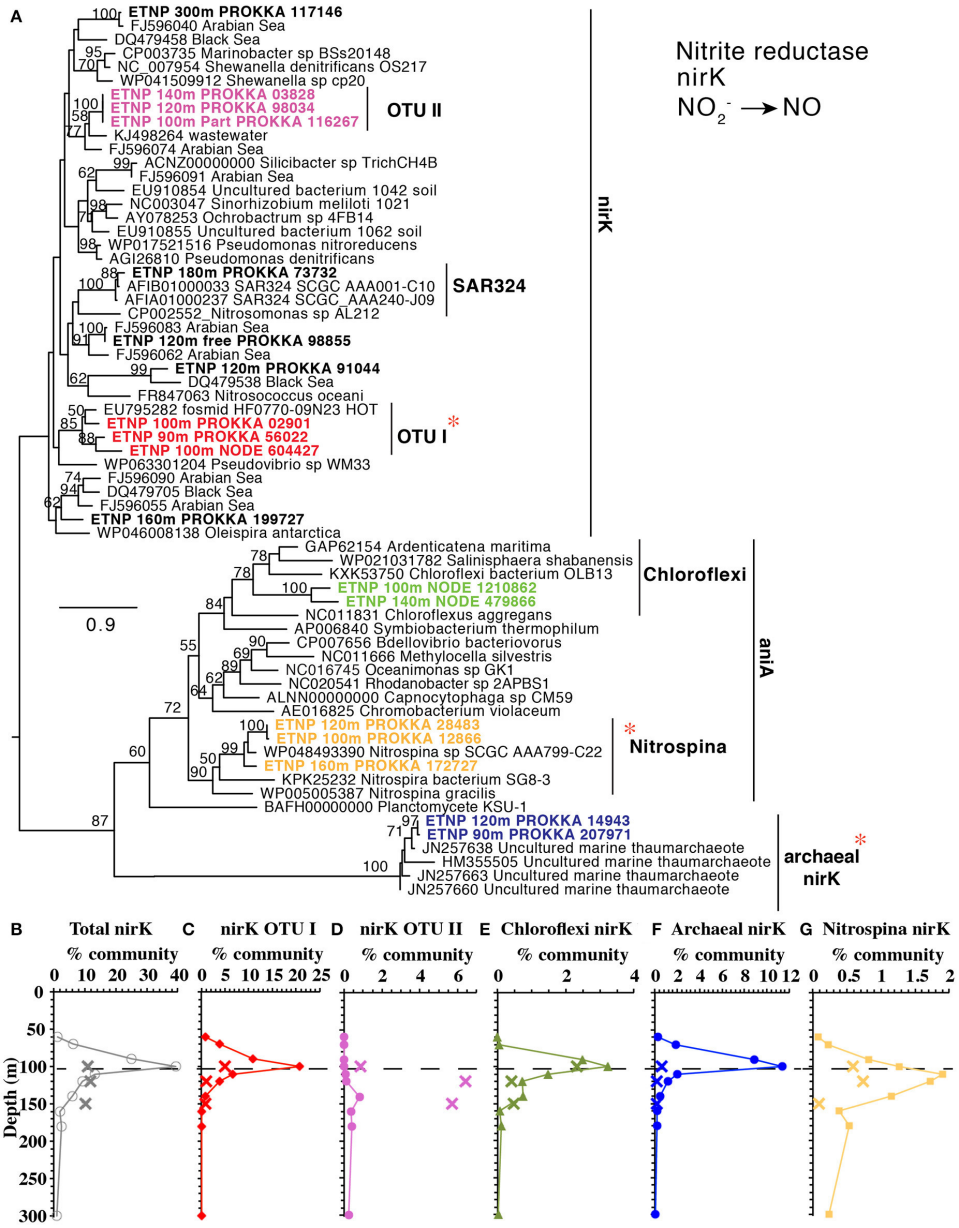
**(A)** Phylogenetic tree of the *nirK/aniA* gene encoding a NO producing nitrite reductase containing copper. Names with ETNP indicate sequences assembled from our metagenomes. Labels colors on the tree match individual OTU depth profiles below **(C–G)**. Depth profiles of **(B)** total *nirK* and *nirK* phylotypes: **(C)** OTU I, **(D)** OTU II, **(E)** Chloroflexi **(F)** ammonia-oxidizing archaea, **(G)** nitrite oxidizer *Nitrospina*. In all depth profiles, Xs indicate particulate (>30 μm) samples. Dashed line represents the top of the ODZ. % Community is calculated in comparison to the single copy core gene RNA polymerase (*rpoB*). Red asterisks indicate placement of ETNP transcripts from Ganesh et al. (2015).

**Figure 6:**
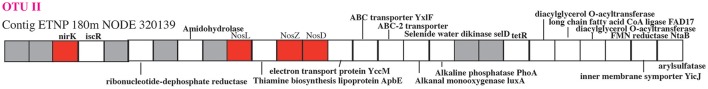
Schematic of a contig containing a *nirK* gene and a *nosZ* gene. Hypothetical proteins are shown in gray and genes of interest are in red. NosL is a copper chaperone and NosD is part of a copper transporter.

The second step in denitrification, nitrite reduction to NO, was dominated by *nirK* (copper containing nitrite reductase) although *nirS* (iron containing nitrite reductase) was also detected in much lower abundances (Figure 1D). *nirK* had a maximum at the top of the ODZ at 100 m, where it was possessed by ~30% of the community (Figure 1D). At least 10 phylotypes were detected and five were present in >1% of the community (Figure 5). Three of these phylotypes, (OTU I, Chloroflexi, and MGI thaumarchaeota) had a maximum in the lower oxycline and were undetectable below 140 m (Figures 5B,D,E), while the *nirK* from nitrite oxidizer *Nitrospina* had maxima within the ODZ at 110 m (Figure 5F). Only one *nirK* phylotype (OTU II) was clearly particle attached and this phylotype also had a maximum in the ODZ (Figure 5C). OTU II *nirK* was found on an assembled contig with a *nosZ* gene (Figure 6), which is discussed with that *nosZ* phylotype below. We note that unlike previous metagenomic examination of *nirK* (Glass et al., 2015; Lüke et al., 2016), MGI Thaumarchaeota were not the dominant *nirK* containing organism. Instead bacterial OTU I was 21% of the community at 100 m (Figure 5). Since transcripts from the 2013 ETNP cruise examined in Glass et al. (2015) place on our OTU I *nirK*, it seems this difference between reports may be methodological rather than due to interannual variability. Unfortunately, assemblies of OTU I *nirK* from our study were short contigs. However, a published SAR11 metagenomic contig from the coastal ETNP contained a partial *nirK* (scaffold 00818) (Tsementzi et al., 2016). This partial SAR11 *nirK* sequence was too short (242 bp) to be a branch on our phylogenetic tree, but aligned with 98.6% identity to the OTU I cluster here using MUSCLE (Edgar, 2004). The published representative of cluster OTU I on our phylogenic tree is from a fosmid obtained Station ALOHA (HF0770-09N23) which also appears to be a SAR11 relative (best BLAST hit for the fosmid using the nt database: SAR11 relative CP003809 HIMB5, E-value: 0.0). Presence in some SAR11 bacteria would explain how OTU I of *nirK* could be so abundant (Figure 5B). While *nirK* was more abundant on particles than in the water column at 120 m (Figure 3), at 100 m, *nirK* was more abundant in bulk water (26%) at station 136 than on particles (11%) at BB2, (Figure S8). This difference is due to the dominance of SAR11 *nirK* OTU I in the water column at the top of the ODZ and is consistent with the free-living nature of SAR11 determined from *rpoB* (Figure 2).

Although *nirS* was present in a much lower percentage of the community than *nirK*, at least five distinct phylotypes were still present (Figure S9). With the exception of anammox *nirS* (Figure 1D), the other 4 phylotypes were present in <2% of the community, and all had maxima within the ODZ at 140 m. Two of these phylotypes (OTU II and III) were particle-attached (Figure S9). The prevalence of the *nirK* copper nitrite reductase over *nirS* is consistent with other untargeted approaches that have also detected *nirK* (Ganesh et al., 2014, 2015; Glass et al., 2015; Lüke et al., 2016) in ODZs but it is at odds with prior primer-based approaches that indicated *nirK* was not environmentally relevant in ODZs (Jayakumar et al., 2013).

#### Nitric oxide reduction

**Figure 7:**
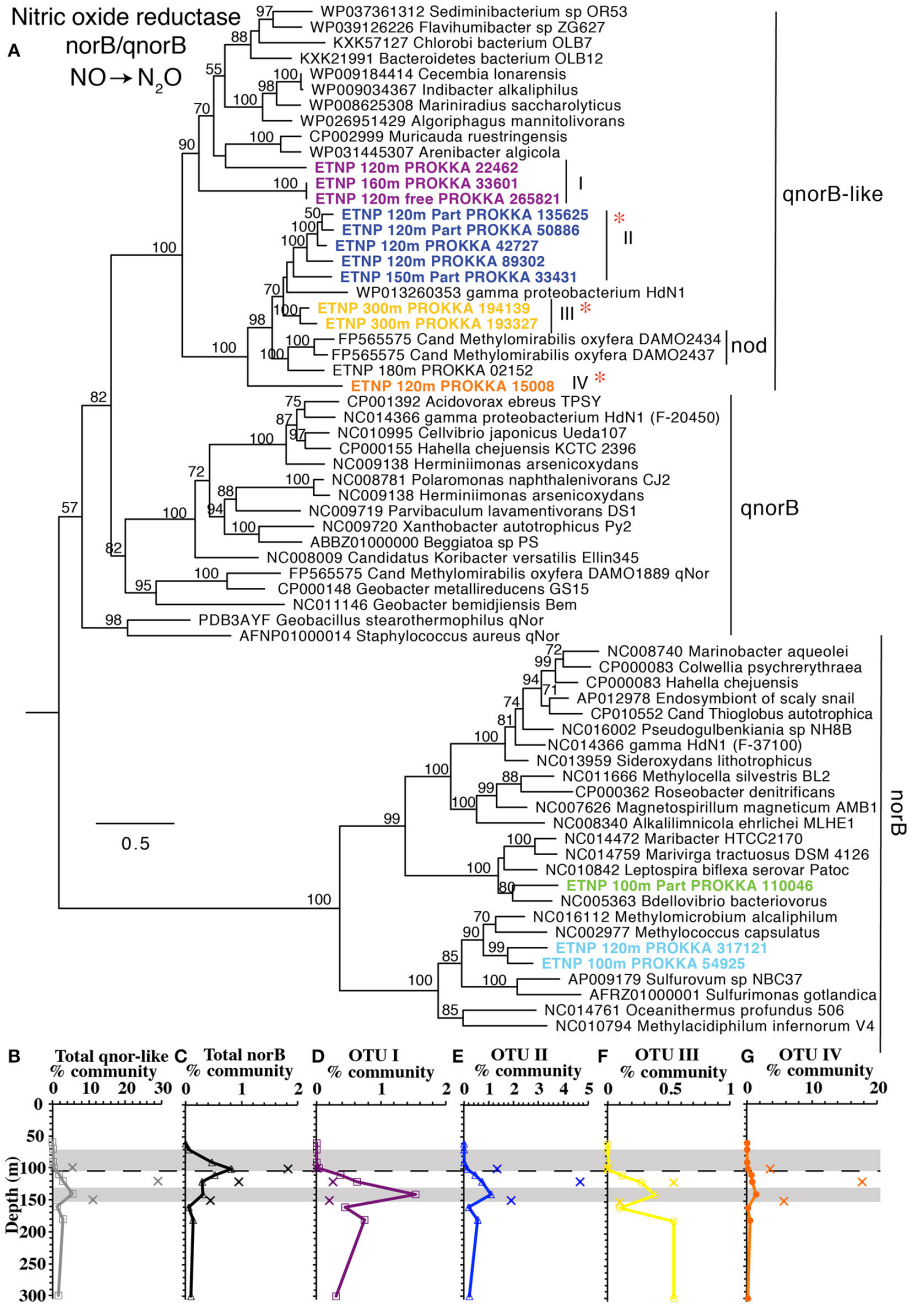
**(A)** Phylogenetic tree of nitric oxide reducing enzymes *norB, qnorB*, and *q-norB-like*. Putative *nod* genes are indicated. Names with ETNP indicate sequences assembled from our metagenomes. Labels and colors on the tree match individual depth profiles **(D–G)**. Depth profiles of **(B)** total *qnorB*-like, and **(C)** total *norB* sequences as well as *q-norB-like* phylotypes: **(D)** OTU I, **(E)** OTU II, **(F)** OTU III, and **(G)** OTU IV. In all depth profiles, Xs indicate particulate (>30 μm) samples. Shaded area indicates the depth of the N_2_O maxima (Figure 1). Dashed line represents the top of the ODZ. % Community is calculated in comparison to the single copy core gene RNA polymerase (*rpoB*). Red asterisks indicate placement of ETNP transcripts from Ganesh et al. (2015).

The third step in denitrification, NO reduction to N_2_O, is mediated by nitric oxide reductase, encoded by genes *norB* or *qnorB* (Lam et al., 2011). However, the canonical forms of these genes were present in very low abundance in this water column (<0.9% *norB*; Figure 1). Instead most of our assembled sequences and short read sequences cluster (Figure 7) with a form of the *qnorB* suggested by *in silico* analysis to be *nod* (nitric oxide dismutase) in the methane-utilizing denitrifier *Cand*. Methylomirabilis from the NC10 phylum, which was theorized to reduce NO straight to N_2_ without a N_2_O intermediate (Ettwig et al., 2012). However, our data is not consistent with all of the genes detected here encoding a nitric oxide dismutase. Many bacteria that have *qnorB* genes in this cluster are not known to dismutate nitric oxide and some also contain nitrous oxide reductase in their genomes (Figure 7). The *qnorB-like* gene has a maximum at 140 m at the same depth as the second N_2_O peak (Figures 1E,F) where N_2_O production rates are modeled to have a maximum (Babbin et al., 2015). However, neither *norB* nor *qnorB* was present at these depths (Figure 1). Theoretically, NO released from a cell could abiotically or non-enzymatically produce N_2_O with iron or thiols under anoxic conditions (Hughes, 2008; Kampschreur et al., 2011; Kozlowski et al., 2016), but this has not been shown in the environment. Thus, if the *qnorB*-like gene is not involved in N_2_O production, there are no known genes present to produce N_2_O in the ODZ. Though we can't rule out the presence of an unknown novel gene for N_2_O production, it would have to be in a completely different gene family from *norB*/*qnorB*/*qnorB*-like to be missed by our methods. When the quinol binding site and active site of *qnor, qnor*-like, putative *nod*, and ETNP assembled contigs are compared, the quinol binding site appears to have more variability between gene types than the active site (Figure S10). While our assembled ETNP sequences do share the differences in the active site seen in the putative nod enzyme, these changes are also seen in the other *qnorB*-like genes (Figure S10). We suggest that at least some, potentially all, of the *qnorB*-like phylotypes detected here retain their function as nitric oxide reductases like their homologs *norB* and *qnorB*.

Sequences for *nod*-like/*qnorB*-like genes from metatranscript assemblies in the coastal ETNP were combined with NC10 16S rRNA sequences related to *Cand*. Methylomirabilis as evidence to suggest a role for methane oxidation in N_2_ production in ODZs (Padilla et al., 2016). The transcripts from the 2013 cruise place on our tree at OTUs II, III, and IV (Figure 7) and assembled sequences from Padilla et al. (2016) belong to our OTU III (Figure S10). In our dataset, however, there is no correlation between any *qnorB*-like gene OTUs and subunits of methane mono-oxygenase, *pmoA*, and *pmoB*. NC10 *pmoA* was possessed by at most of 0.1% of the community, and *pmoB* was barely above detection and no NC10 *pmoB* was detectable in our metagenomes. In contrast, the *qnorB*-like gene was present in >25% of the community in particles (Figure 3). Thus, the large difference in % community between gene in these two pathways makes it seems unlikely that the abundant organisms containing the *qnorB*-like gene are involved in methane oxidation. Additionally, when our long contigs containing *qnorB-like* gene are examined, none of the contigs had BLAST hits in National Center for Biotechnology Information (NCBI) nucleotide collection database (nt) (NCBI Resource Coordinators, 2017) with E values <−20. Contig 120 m particle NODE 148559 has competence genes right next to the *qnorB-like* gene, implying that *qnorB-like* gene could have been transferred (Figure S11), and in general, the contigs appear to be dissimilar to each other, even between multiple contigs in the same *qnorB*-like phylotype (OTU II; Figure S11). Thus, the *qnorB*-like gene appears to be in multiple organisms in the ETNP, which are not related to *Cand*. Methylomirabilis or other bacteria in the NCBI nucleotide database.

#### N_2_ gas production

**Figure 8:**
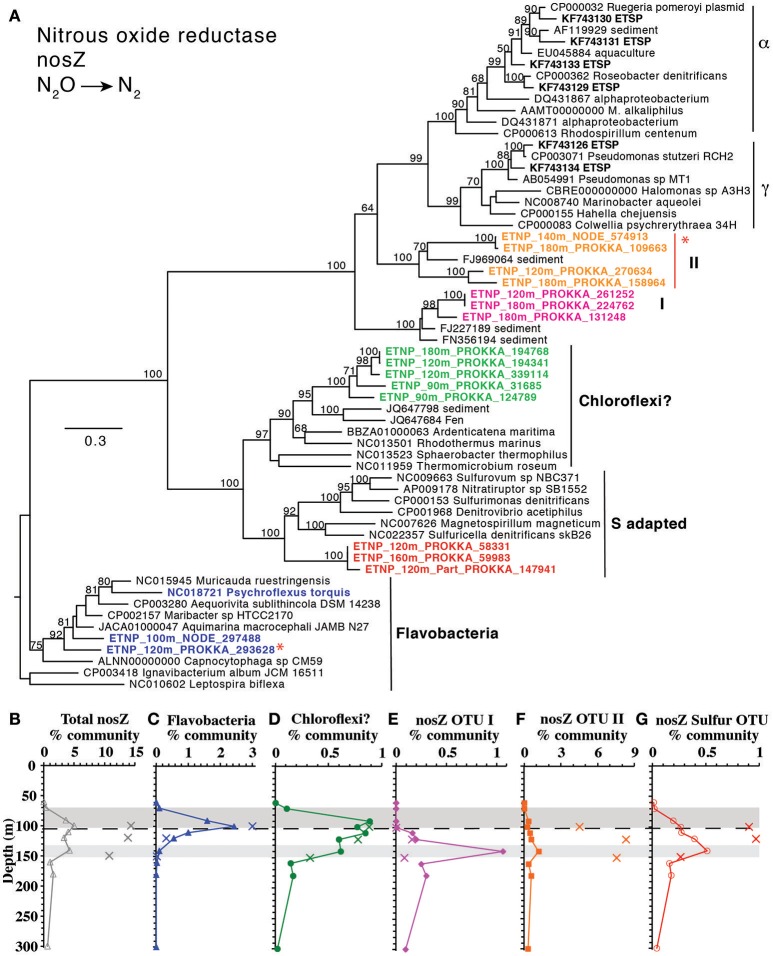
**(A)** Phylogenetic tree of N_2_O reductase enzyme *nosZ*. Names with ETNP indicate full-length sequences assembled from our metagenomic samples. Colors on the tree match individual OTU depth profiles **(C–G)**. Depth profiles of **(B)** total *nosZ* and of *nosZ* phylotypes: **(C)** Flavobacteria, **(D)** Chloroflexi, **(E)** OTU I, **(F)** OTU II, and **(G)** Sulfur adapted. In all depth profiles, Xs indicate particulate (>30 μm) samples. Shading on depth profiles indicates depth of the two N_2_O maxima (Figure 1). Dashed line represents the top of the ODZ. % Community is calculated in comparison to the single copy core gene RNA polymerase (*rpoB*). Red asterisks indicate placement of ETNP transcripts from Ganesh et al. (2015).

The final step in denitrification, N_2_ production from N_2_O, is mediated by *nosZ*, which was elevated from 90 to 140 m (Figure 1G). The *nosZ* gene depth profile had two maxima at 100 and 140 m (Figure 1), which corresponded to the two N_2_O concentration maxima in the lower oxycline and at 140 m. Potential N_2_O reduction rates also had a maximum at 140 m (2.7 nM/d, Babbin et al., 2015). The N_2_O reductase gene *nosZ* had 5 phylotypes in the ETNP. Notably these ETNP *nosZ* phylotypes were completely different from sequences from the coastal ETSP (Castro-González et al., 2015) (Figure 8A). ETNP N_2_O reductase (*nosZ*) phylotypes could be separated into two groups based on their depth distribution with maxima corresponding to the two N_2_O concentration maxima (Figure 8). It is possible that these different depth profiles represent different tolerances of the organisms to oxygen. The Flavobacterial *nosZ* was abundant at the top of the ODZ with a maximum at 100 m, corresponding to the upper *nosZ* gene maxima (Figure 8B). This group is represented by one extensive 11 gene contig (ETNP 120 m NODE 73975) containing *nosD, nosZ*, and cytochrome c (Figure S12). The best BLAST hit for this contig was the Flavobacteria *Lutibacter* sp. LPI (#CP013355), confirming the Flavobacteria affinity of this *nosZ*. The other 4 phylotypes represent novel clades. One clade of *nosZ* sequences clustered with *Chloroflexi*, but this clade was not represented by multi-gene contigs. The Flavobacteria and potential *Chloroflexi* phylotypes both had their maxima at the upper N_2_O peak and made up roughly similar % community in the water and on particles (Figures 8B,C, Figure S13). The other three unidentified phylotypes each had maxima at the second N_2_O maximum at 140 m (Figures 8D–F). One of the nosZ phylotypes had the C terminal extension typical of S oxidizing bacteria, suggesting it may belong to an autotrophic denitrifier, and this phylotype was enriched in particles (Figures 8A,F, Figure S13). This clade was represented by a contig containing 23 gene sequences (ETNP 120 m NODE 137405) including a photosystem I gene *psaC* along with *nosL, nosD*, and *nosZ* (Figure S12). The fourth *nosZ* phylotype, OTU I, was predominately free-living (Figure 8D, Figure S13). This phylotype was represented by three extensive contigs (Figure S12). Multiple genes on the contigs identified this organism as a heterotroph, including beta-galactosidase. Particles were dominated by the fifth *nosZ* phylotype, OTU II (Figure 8E, Figure S13). Two extensive contigs represent the upper branch of OTU II (Figure S12). Along with genes for *nosL, nosD*, and *nosZ*, contig ETNP 180 m NODE 320139 also contained a *nirK* gene belonging to *nirK* OTU II, a phylotype that was also particle attached. Thus, *nosZ* phylotype OTU II and *nirK* OTU II are both the same unidentified organism (Figure 6).

**Figure 9:**
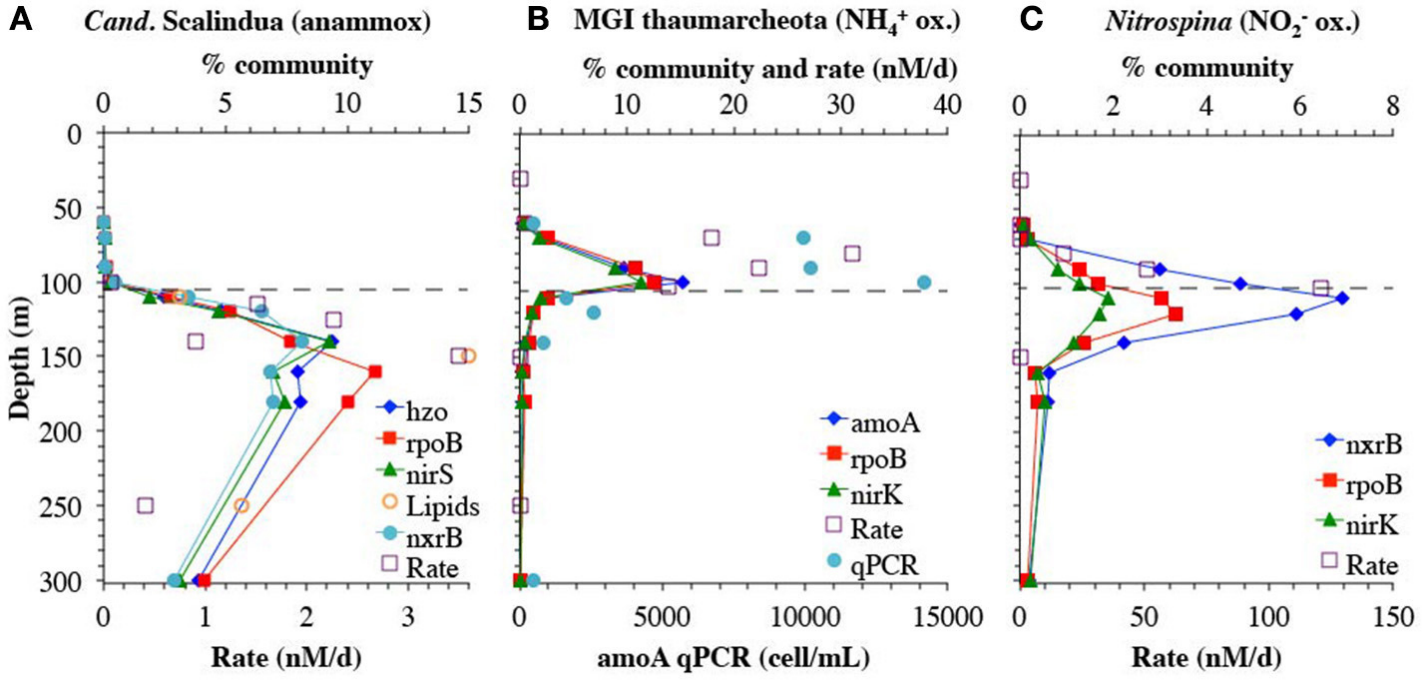
A comparison between genes and rates. **(A)** Genes for anammox bacteria *Cand*. Scalindua for nitrite reductase (*nirS*), hydrazine oxidoreductase (*hzo*) and single copy core gene *rpoB*. Anammox rates are calculated by subtracting N_2_O reduction rates (Babbin et al., 2015) from total N_2_ production rates from BB2 (Babbin et al., 2014). PC-monoether ladderane lipid values are from St 136 in Sollai et al. (2015), but are normalized to fit on the % community axis; values are 15 pg/L at 110 m, 72 pg/L at 150 m, 27 pg/L at 250 m, and 25 pg/L at 350 m. **(B)** genes for ammonia oxidizing MGI Thaumarcheota for ammonia monooxygenase (*amoA*), nitrite reductase (*nirK*), and single copy core gene *rpoB*. Ammonia oxidizing rates and *amoA* qPCR are from BB2 in Peng et al. (2015). **(C)** genes from nitrite oxidizing bacterium *Nitrospina* for nitrite reductase (*nirK*), nitrite oxidoreductase (*nxrB*), and single copy core gene *rpoB*. Nitrite oxidizing rates are from BB2 in Peng et al. (2015). Dashed line represents the top of the ODZ. % Community is calculated in comparison to the single copy core gene RNA polymerase (*rpoB*).

Genes representing the second N_2_ producing pathway, anammox, had a maximum from 120 to 180 m (Figures 2D,G, 9A). Notably the depth profile of the gene for hydrazine oxidoreductase (*hzo*), the final step in the anammox pathway, was consistent with both the *nirS* and *rpoB* reads that branched with *Cand*. Scalindua (Figure 9A). As expected, only one phylotype of anammox bacteria was present on both *hzo* and *nirS* phylogenetic trees (Figures S9, S14). The combined maxima in anammox genes and *nosZ* corresponded to the upper N_2_ gas maximum (Figure 1H). This depth profile for anammox genes is consistent with intact ladderane lipid data from the same station (Sollai et al., 2015) and with calculated anammox rates (Figure 9A). Anammox rates were estimated by subtracting N_2_O reduction rates (Babbin et al., 2015) from total N_2_ production rates from BB2 (Babbin et al., 2014) and these differential rates showed some variability, but still had a maximum at the same depth as the metagenomic reads (Figure 9A).

### Correlation of functional gene abundance with activity

Although gene presence assessed by metagenomics only confirms the potential for a given function, the anammox data suggest a close correspondence in space between the presence of an organism and its activity in this environment. We further assessed this relationship in two other well-characterized microbial groups, the ammonia-oxidizing MGI Thaumarchaeota, and the nitrite oxidizer *Nitrospina*. The depth profile of the ammonia monooxygenase gene *amoA* was tightly correlated with MGI Thaumarchaeota specific *nirK* and *rpoB* reads, and all three genes had a maximum at 100 m (Figure 9B). Previously determined qPCR measurements of *amoA* at BB2 also had a maximum at 100 m (Peng et al., 2015), and the overall depth profiles were consistent with a slope of 1106.2 cells per mL/% community with *amoA* (*R*^2^ = 0.7). Rates of ammonia oxidation, which were calculated including ammonia oxidized to both nitrite and nitrate (Peng et al., 2015) had a maximum at 80 m, above the maxima in both qPCR and metagenomic reads (Figure 9B).

The gene encoding the nitrite oxidizing enzyme nitrite oxidoreductase (*nxrB*) had a maximum in the ODZ at 110 m, again consistent with *Nitrospina* specific *nirK* and *rpoB* reads. Unlike in the Arabian Sea (Lüke et al., 2016), here nitrite oxidoreductase can be solely attributed to *Nitrospina* (Figure 9C, Figure S6). Like isolate *Nitrospina gracilis* (Lücker et al., 2013), it appears that the ODZ *Nitrospina* has two copies of the *nxrB* gene per genome, as determined by comparison with the single copy core gene *rpoB* (Figure 9C). This genetic potential for nitrite oxidation within the ODZ is consistent with measured nitrite oxidation rates of >100 nM/d at BB2 (Figure 9C) (Peng et al., 2015) and nitrite oxidation rates had a similar depth profile as the metagenomic read depth profile for *Nitrospina*. Nitrite oxidizers in the ETSP had a high affinity for oxygen with a low oxygen K_m_ of 0.5 ± 4.0 nM (Bristow et al., 2016). It has been suggested that oxygen production by *Prochlorococcus* in the ODZ can fuel nitrite oxidation (Garcia-Robledo et al., 2017).

The three examples above, *Cand*. Scalindua, MGI Thaumarchaeota, and *Nitrospina*, demonstrate that the read placement technique produces consistent results when applied to multiple genes in the same organism, supporting the use of normalized read placement as a semi quantitative method to examine microbial depth profiles. This approach is also valuable for understanding distributions of as yet uncharacterized organisms. For example, previously, the phylogeny of metagenomic and metatranscriptomic reads in ODZs have been identified with BLAST using the NCBI nucleotide collection database (nt/nr) (Ganesh et al., 2014, 2015; Glass et al., 2015). When re-examining this previous data with our read placement approach that incorporates the corresponding metagenome assemblies on the reference tree, we find the same functional gene identification (*narG, nirK* etc) as found with BLAST, but the phylogenetic affiliation of each gene can be better determined. BLAST identifications depend greatly on the composition of the database (Fuchsman and Rocap, 2006), and is a determination via local alignment. The placement approach can provide a higher resolution determination among closely related organisms in part because the whole sequence is used for comparison to a known reference (Berger et al., 2011). For example, using BLAST against NCBI nt/nr database, the phylogenetic identity of nitric oxide reductase transcripts from the coastal ETNP in 2013 was highly variable with depth (Ganesh et al., 2015). However, here these transcripts consistently place on three uncultured ETNP *qnor*-like OTUs assembled from our metagenome, which were not present in the original NCBI nt/nr BLAST database (Figure 7). Similarly, the identity of N_2_O reductase *nosZ* transcripts from this same ETNP metatranscriptome were reported as predominantly unknown prokaryote (Ganesh et al., 2015). Read placement of those transcripts on a phylogenetic tree indicates that the unknown prokaryote transcripts are all OTU II (Figure 8), which can now be linked to other N cycling genes (Figure 6) through our assembly and to a particle-attached lifestyle (Figure 8). Thus, read placement techniques in combination with assemblies from the relevant environment allow us to take the next step in understanding the community in ODZs.

### N_2_O and N_2_ production in the oxycline

We apply this holistic approach to analysis of N cycling genes with chemical measurements and rate data to the oxycline above the ETNP ODZ, which has been implicated as a potential source of N_2_O, a potent greenhouse gas, to the atmosphere (Cohen and Gordon, 1978; Yamagishi et al., 2007; Babbin et al., 2015). Here, concentrations of N_2_O were highest (106 nM) at 90 m within the oxycline (Figure 1F, Peng et al., 2015). In addition to production by nitric oxide reductases during denitrification, N_2_O is also produced by ammonia oxidizing archaea (Santoro et al., 2011). The enzymes involved in this process are unclear and the last step of N_2_O production may be non-enzymatic in archaea (Kozlowski et al., 2016). From isotopomer analysis of N_2_O in the upper ETNP oxycline, ammonia oxidizers contribute more than denitrifiers to N_2_O production, but in the lower oxycline both processes may be present (Yamagishi et al., 2007). Indeed, in the ETSP incubation experiments indicated that both ammonia oxidation and denitrification contributed to N_2_O production in the lower oxycline (Ji et al., 2015). Here, at station BB2, ammonia oxidation rates, which were solely attributed to archaea, were measurable throughout the oxycline, corresponding with the N_2_O maximum, and were still 13 nM/d at 103 m (Peng et al., 2015). This is consistent with our metagenomic data indicating MGI Thaumarchaeota made up to 12% of the community at 100 m, corresponding to the upper N_2_O maximum (Figures 2E,F, 5). However, if ammonia-oxidizing archaea were the only source of N_2_O here, the by-product N_2_O would be ~20% of oxidized ammonia in the oxycline at BB2 (Peng et al., 2015). N_2_O yields of marine archaea under normal oxygen conditions are <1% (Santoro et al., 2011; Loscher et al., 2012) though yields up to 1.6% were found in the oxycline of the ETSP (Ji et al., 2015). Thus, some form of denitrification in the lower oxycline (90–100 m) is necessary to explain the N_2_O measurements (Babbin et al., 2015; Peng et al., 2015). However, here no nitric oxide reductases (either *norB* or *qnorB-like*) were detected in the water column at 90 m and *norB* was only present in 1% of the community at 100 m in our whole water samples (Figure 1). We suggest production of N_2_O inside particles can explain these N_2_O measurements. Although *qnorB-like* nitric oxide reductase was not detected in the water column in the oxycline at station 136, it was present in particles (5.5% of the particle community) at the base of the oxycline (100 m) at station BB2 and *norB* was also present (2.2% of the particle community) (Figure S8). Thus, particle communities in the oxycline have the capacity to produce N_2_O. This is understandable as there are oxygen gradients inside marine organic particles (Ploug et al., 1997), and measurements of diatom aggregates indicate that at ~100 μmol L^−1^ ambient O_2_ and below aggregates contained anoxic regions from which N_2_O and N_2_ production could be measured (Stief et al., 2016). Zooplankton carcasses may also be sites of denitrification below ~10 μmol L^−1^ ambient O_2_ (Stief et al., 2017). Thus, based on the measured STOX oxygen concentrations (4.7 and 0.8 μM at 90 and 100 m) the majority of particles should contain anoxic zones in the ETNP lower oxycline. N_2_O production from sediment trap material under oxic conditions has been shown previously in the North Pacific (Wilson et al., 2014).

N_2_O is also potentially consumed in the lower oxycline, forming N_2_. Although denitrification can be 50% inhibited by 200–300 nM O_2_ (Dalsgaard et al., 2014), N_2_ production in the oxycline is consistent with the N_2_ gas concentration profile (Figure 1H). Potential N_2_O reduction rates extend into the oxycline (1 nM/d at 85 m), though vials for these rates were incubated anaerobically (Babbin et al., 2015). Importantly, measured gas in the water column, either N_2_O or N_2_, represents both water column and particulate production. As with N_2_O production, particles may also be important for N_2_ production in the oxycline. N_2_O reductase (*nosZ*) was found in 3–5% community in bulk water in the oxycline at station 136 (Figure 1G), and only two phylotypes were present (*Flavobacteria* and *Chloroflexi*) (Figures 8B,C). This suggests that all denitrifiers may not have the same oxygen inhibition threshold or that *Flavobacteria* and *Chloroflexi* are facultative denitrifiers. Notably, *nosZ* was also enriched in the 100 m particles at station BB2 where it was contained by 13% of the community (Figure S8) represented by *nosZ* OTU II and Flavobacteria *nosZ* (Figure 8). Thus, much of the N_2_ gas production in the oxycline may occur on particles.

### Niche partitioning

Microbes containing N cycling genes examined here separate into three groups with distinct niches: particle-attached, free-living with tolerance to low levels of oxygen, free-living with preference to complete anoxia. Genes in bacteria attached to particles could either be taking advantage of the abundant organic matter there or of the more reduced conditions found inside particles. It appears that three phylotypes of *narG*, one phylotype of *nirK*, two phylotype of *nirS*, two phylotype of *qnor*-like and two phylotype of *nosZ* genes are particle attached, including the *nosZ* affiliated with S-oxidizers (Figures 4, 5, 7, 8, Figures S9, S13). In the water column, genes can be separated into two niches based on oxygen content. Genes found in the upper ODZ are likely exposed to nanomolar oxygen either by mixing or by O_2_ production by ODZ *Prochlorococcus* (Garcia-Robledo et al., 2017). Some OTUs appear to be tolerant of oxygen while others are only abundant below 120 m after all possible O_2_ has been removed. Genes from oxygen utilizing microbes, ammonia oxidizing archaea and nitrite oxidizing bacteria were found in the upper ODZ (Figure 9). Genes for anammox bacteria were abundant below 120 m (Figure 9), which could imply a lack of tolerance to oxygen or competition with ammonia and nitrite oxidizers for its reactant ammonium (Penn et al., 2016). All of the *nirK* phylotypes that are enriched in bulk water are found in the upper ODZ while all the *nirS* OTUs are abundant below 120 m (Figure 5, Figure S9), implying different oxygen tolerances for bacteria with these genes. Additionally, all the *qnor*-like genes were abundant below 120 m (Figure 7). Two water column *nosZ* phylotypes were found in the upper ODZ while one free-living *nosZ* was abundant below 120 m (Figure 8, Figure S13), indicating diversity of oxygen tolerance between types of denitrifiers. The free-living SAR11 *narG* phylotypes were abundant throughout the ODZ, but not in the oxycline, so did not clearly fall into our defined water-column niches (Figure 4, Figure S13). Due to their differing depth profiles and niches, it is possible that not all these free-living microbes have all the genes necessary to perform complete denitrification. In all, these data highlight the diversity of N reducing microbes in the ODZ.

### Synthesis

Here we describe gene abundances and distinct phylotypes for all key steps in the low oxygen N cycle in an open-ocean ODZ community. Although gene presence only indicates potential activity, relative gene abundances here correlated closely with features in chemical profiles and measured rates for denitrification, anammox, ammonia oxidation, and nitrite oxidation (Babbin et al., 2014, 2015; Peng et al., 2015). Both anammox and N_2_ producing denitrifiers were present in the water column with denitrifiers found at shallower depths, reaching the oxycline. Overall, denitrification genes, with the exception of nitrate reductase, were enriched in >30 μm particles. However, when examined at a phylotype level, two N_2_O reductase phylotypes were enriched on particles while three other *nosZ* phylotypes were actually not enriched in particles. Furthermore, *Flavobacteria* and *Chloroflexi nosZ* phylotypes had maxima within lower oxycline, while the remaining *nosZ* phylotypes had maxima within the ODZ. These data highlight the diversity of denitrifiers both phylogenetically and potentially functionally.

While we did not find evidence for denitrification without an N_2_O intermediate by autotrophic methane-oxidizers, autotrophic denitrification is still a distinct probability in the ETNP. The presence of a C-terminal extension on *nosZ* in a group of assembled contigs indicated the presence of a S-oxidizing autotrophic denitrifying phylotype on particles. A S-oxidizing autotrophic denitrifier was previously found on particles in the suboxic zone of the Black Sea (Fuchsman et al., 2012a). These data support the possibility of low level but widespread S cycling in particles under low oxygen conditions.

The largest N_2_O maxima in the ETNP are in the oxycline above the ODZ. Since ammonia oxidation rates are too low to support all the N_2_O production in the oxycline (Peng et al., 2015), an additional source of N_2_O production is likely (Babbin et al., 2015). Our metagenomic data supports production of N_2_O inside particles in the oxycline, rather than denitrification in the water column.

In a warming ocean with lower oxygen concentrations, the area of both ODZs and the oxycline above them may expand. Understanding the diversity and function of water column and particle associated communities in these regions may be critical for correctly predicting the magnitude of N loss and N_2_O release to the atmosphere.

## Author contributions

CF: collected the samples, created metagenomic libraries, designed, and performed analyses and wrote the paper; JS: wrote code essential to the analyses and edited the paper; CM: assembled the metagenomes, wrote code used in analyses, and edited the paper; AD and GR: helped design analyses and edited the paper.

### Conflict of interest statement

The authors declare that the research was conducted in the absence of any commercial or financial relationships that could be construed as a potential conflict of interest.
