# Supplementary material for: Niche Partitioning of the N Cycling Microbial Community of an Offshore Oxygen Deficient Zone
Source: Front Microbiol. 2017 Dec 5;8:2384. doi: 10.3389/fmicb.2017.02384 (PMC5723336; doi:10.3389/fmicb.2017.02384)
Supplement: Supplementary file 1 [file Image1.PDF]

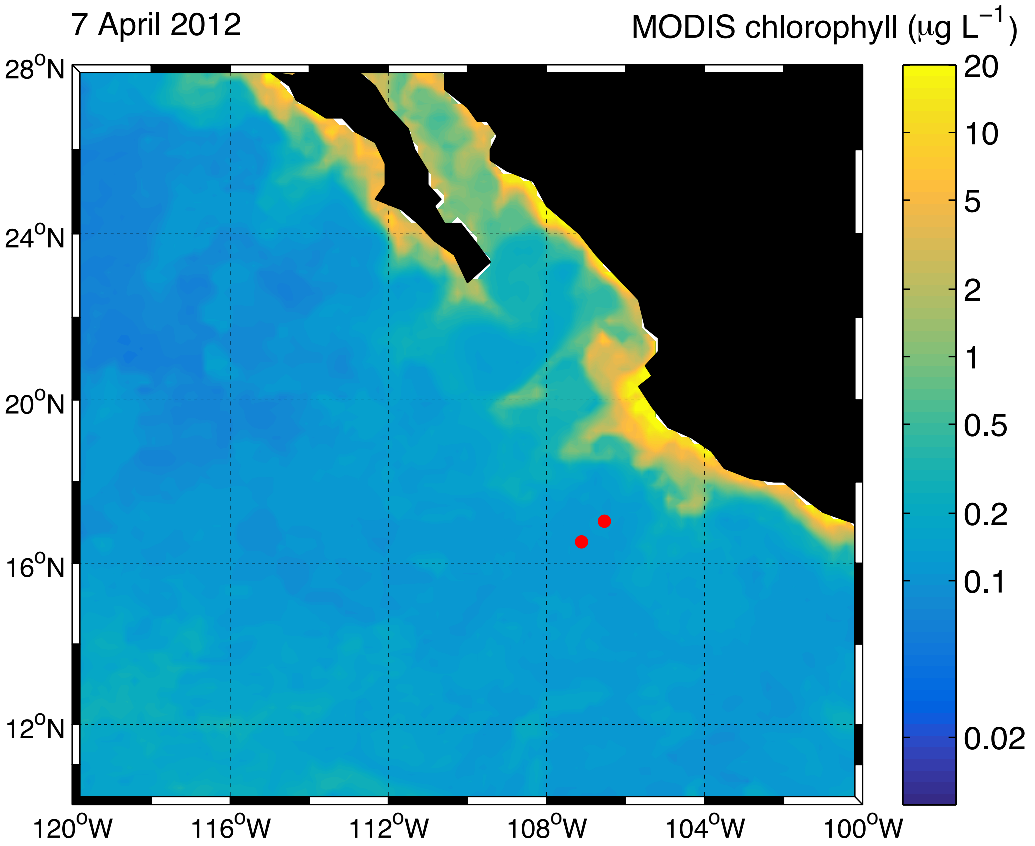

Figure S1. Map of 8 day averaged satellite chlorophyll starting on April 7, 2012, which includes the time period of metagenomic sampling (April 8 and 9). Red dots indicate stations sampled for metagenomics.
