# Supplementary material for: Niche Partitioning of the N Cycling Microbial Community of an Offshore Oxygen Deficient Zone
Source: Front Microbiol. 2017 Dec 5;8:2384. doi: 10.3389/fmicb.2017.02384 (PMC5723336; doi:10.3389/fmicb.2017.02384)
Supplement: Supplementary file 2 [file Image2.PDF]

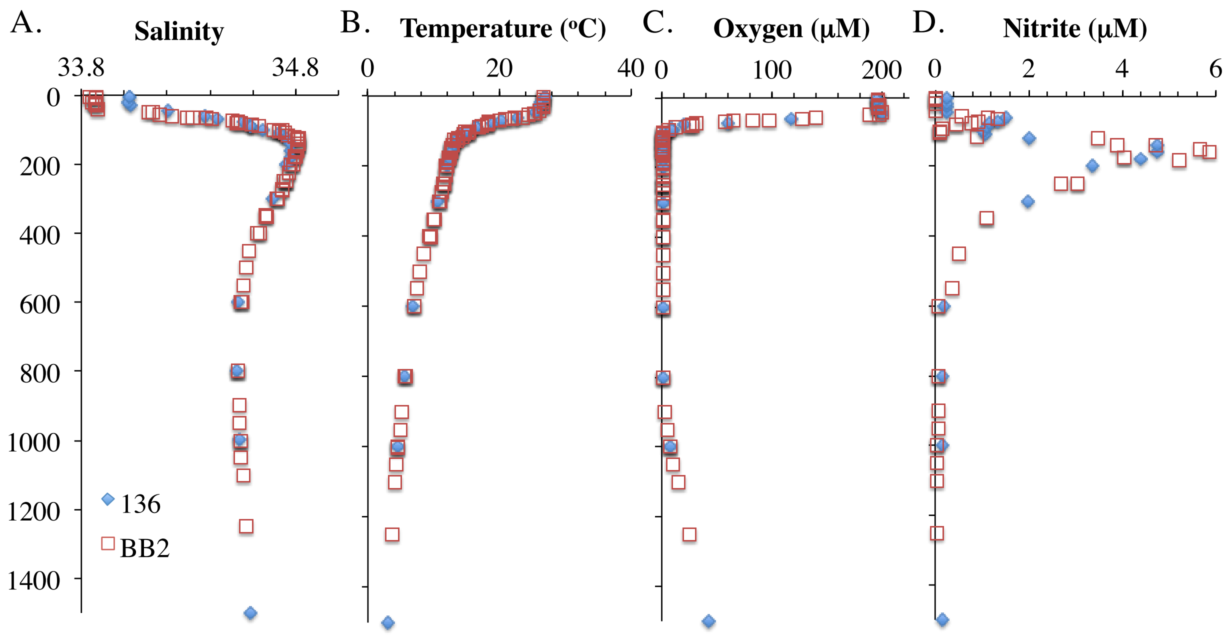

Figure S2. A comparison of station 136 and BB2 with the parameters: A) salinity, B) temperature, C) oxygen and D) nitrite.
