# Supplementary material for: Niche Partitioning of the N Cycling Microbial Community of an Offshore Oxygen Deficient Zone
Source: Front Microbiol. 2017 Dec 5;8:2384. doi: 10.3389/fmicb.2017.02384 (PMC5723336; doi:10.3389/fmicb.2017.02384)
Supplement: Supplementary file 4 [file Image4.PDF]

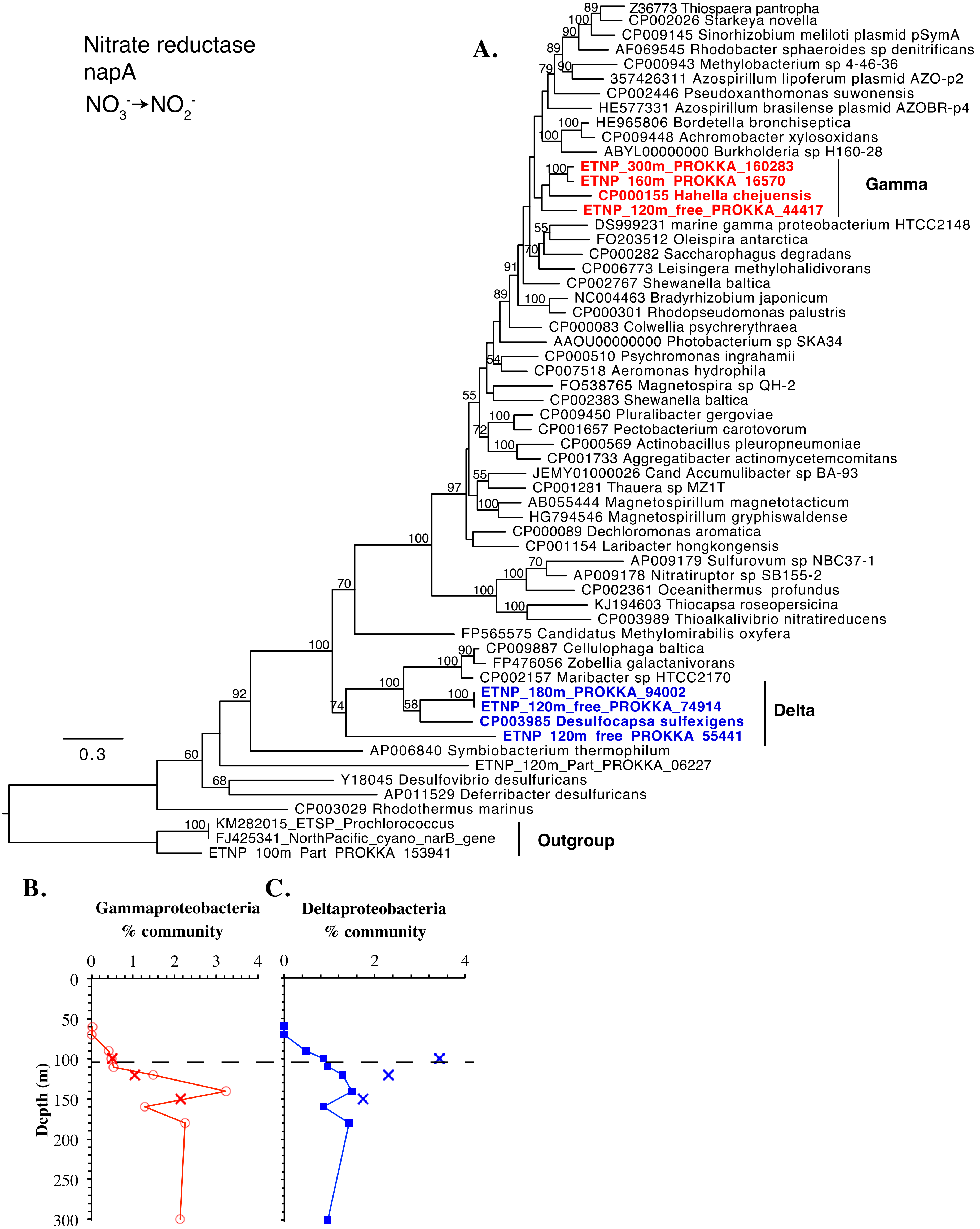

Figure S4. A) Phylogenetic tree of genes for the periplasmic nitrate reductase *napA*. Names with ETNP indicate sequences assembled from our metagenomic samples. Labels and colors on the tree match phylotype depth profiles (B) deltaproteobacteria and (C) gammaproteobacteria. In all depth profiles, Xs indicate particulate (>30 µm) samples. Dashed line indicates the top of the ODZ. % Community is calculated in comparison to the single copy core gene RNA polymerase (*rpoB*).
