# Supplementary material for: Niche Partitioning of the N Cycling Microbial Community of an Offshore Oxygen Deficient Zone
Source: Front Microbiol. 2017 Dec 5;8:2384. doi: 10.3389/fmicb.2017.02384 (PMC5723336; doi:10.3389/fmicb.2017.02384)
Supplement: Supplementary file 5 [file Image5.PDF]

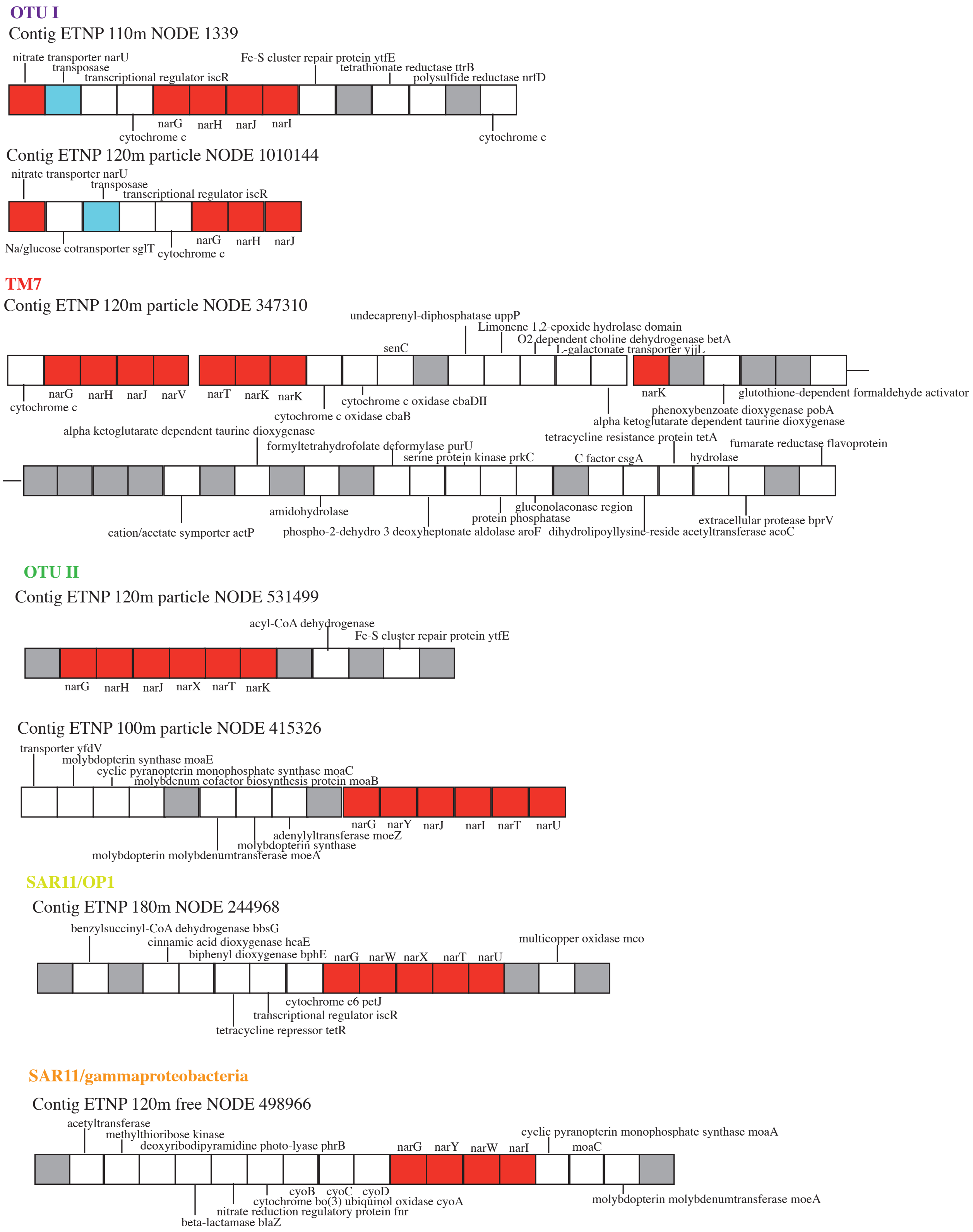


Figure S5. Schematic of selected contigs containing nitrate reductase gene *narG*. Hypothetical proteins are shown in gray. Proteins related to nitrate reduction are shown in red. Contigs are clustered by their phylogenetic affiliation.
