# Supplementary figures and images for: Niche Partitioning of the N Cycling Microbial Community of an Offshore Oxygen Deficient Zone
Source: Front Microbiol. 2017 Dec 5;8:2384. doi: 10.3389/fmicb.2017.02384 (PMC5723336; doi:10.3389/fmicb.2017.02384)

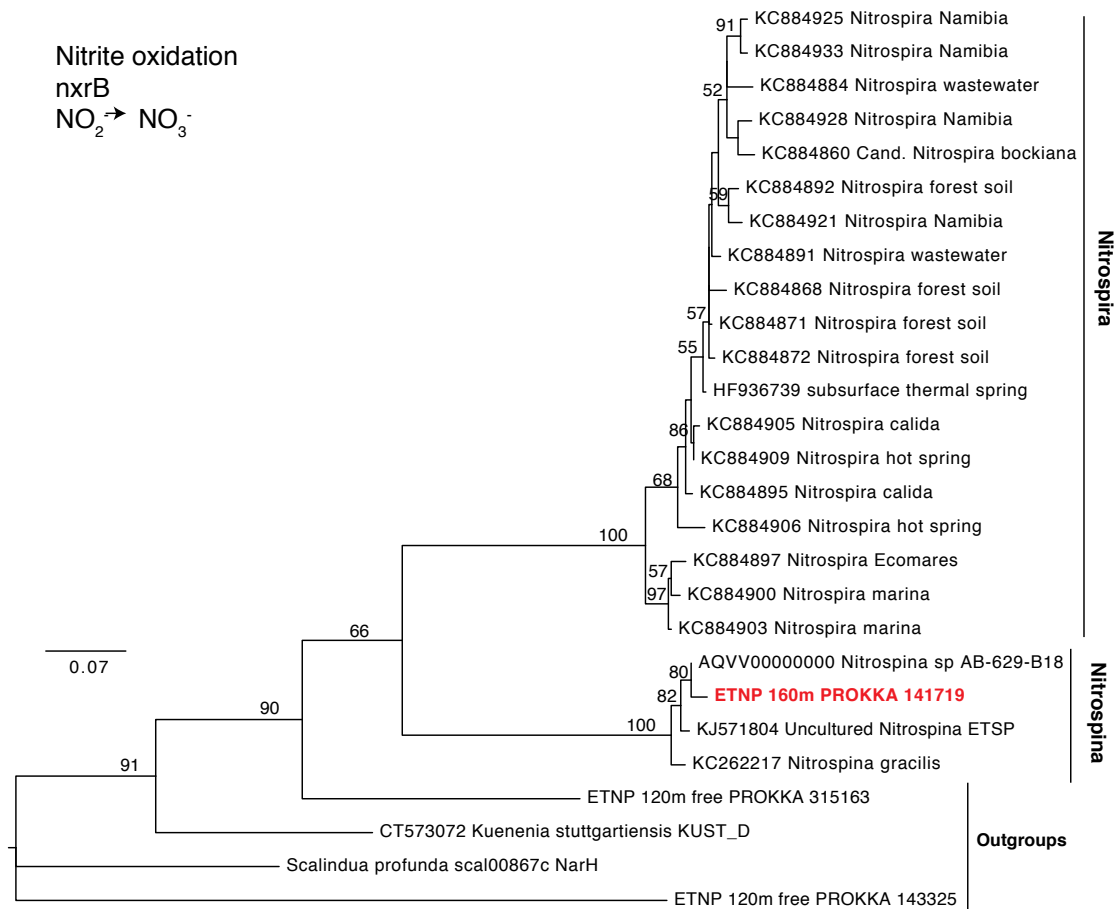

5

Supplement: Supplementary file 6 [file Image6.PDF]
