# Supplementary material for: Niche Partitioning of the N Cycling Microbial Community of an Offshore Oxygen Deficient Zone
Source: Front Microbiol. 2017 Dec 5;8:2384. doi: 10.3389/fmicb.2017.02384 (PMC5723336; doi:10.3389/fmicb.2017.02384)
Supplement: Supplementary file 7 [file Image7.PDF]

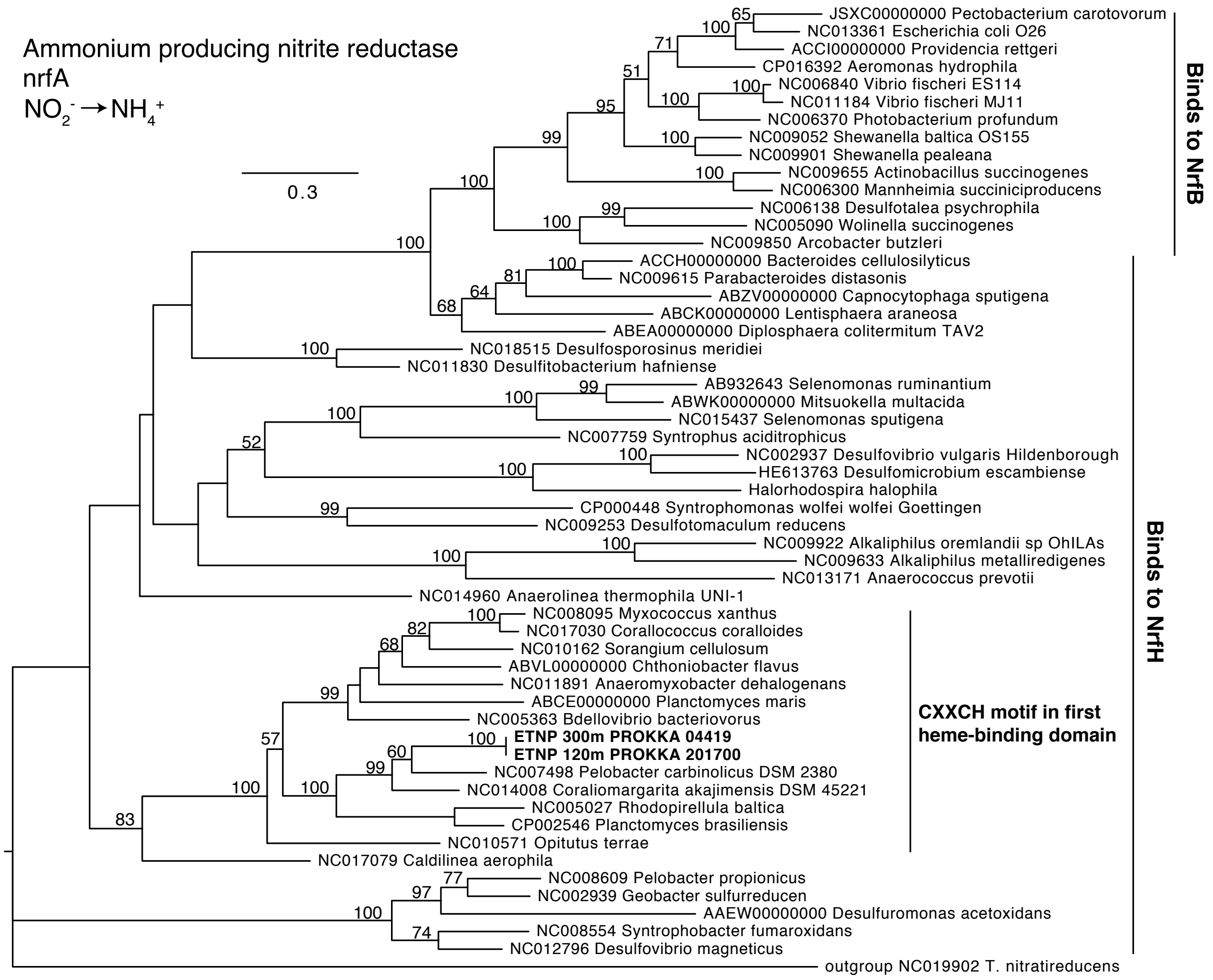

Figure S7. Phylogenetic tree of genes for ammonia producing nitrate reductase *nrfA*. Names with ETNP indicate sequences assembled from our metagenomic samples. Information on tree is based on Welsh et al (2014) AEM 80: 2110-2119. ETNP sequences are in a cluster with an unusual heme-binding domain.
