# Supplementary material for: Niche Partitioning of the N Cycling Microbial Community of an Offshore Oxygen Deficient Zone
Source: Front Microbiol. 2017 Dec 5;8:2384. doi: 10.3389/fmicb.2017.02384 (PMC5723336; doi:10.3389/fmicb.2017.02384)
Supplement: Supplementary file 8 [file Image8.PDF]

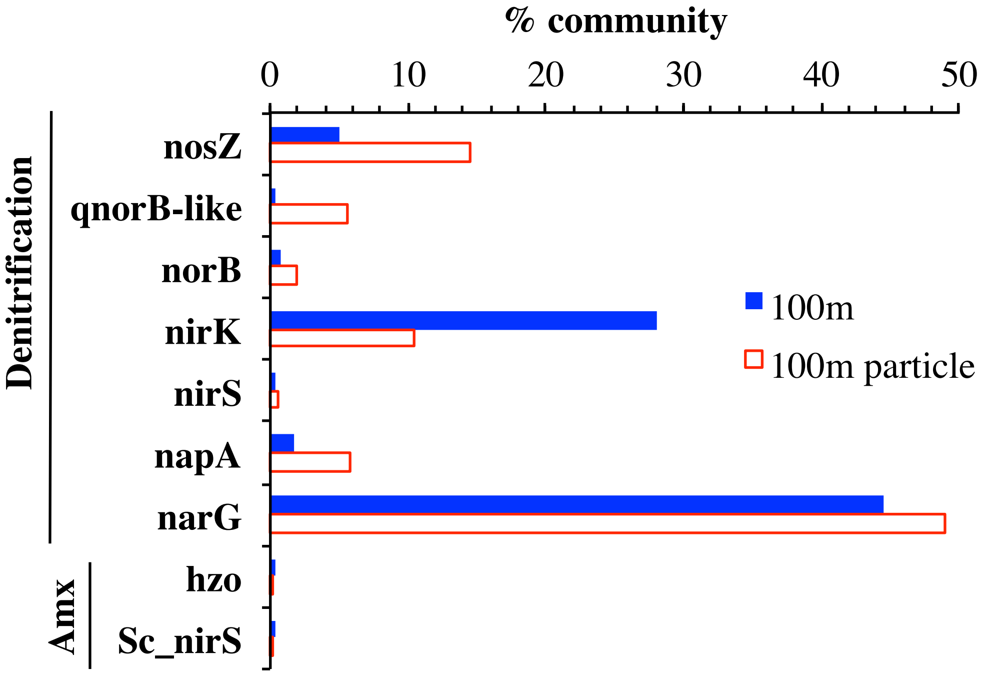

Figure S8. A comparison of denitrification and anammox (Amx) genes from bulk water from 100m (St 136) and >30 µm particle from 100m (St BB2). Sc-nirS indicates anammox (Scalindua) *nirS*. % Community is calculated in comparison to the single copy core gene RNA polymerase (*rpoB*).
