# Supplementary material for: Niche Partitioning of the N Cycling Microbial Community of an Offshore Oxygen Deficient Zone
Source: Front Microbiol. 2017 Dec 5;8:2384. doi: 10.3389/fmicb.2017.02384 (PMC5723336; doi:10.3389/fmicb.2017.02384)
Supplement: Supplementary file 9 [file Image9.PDF]

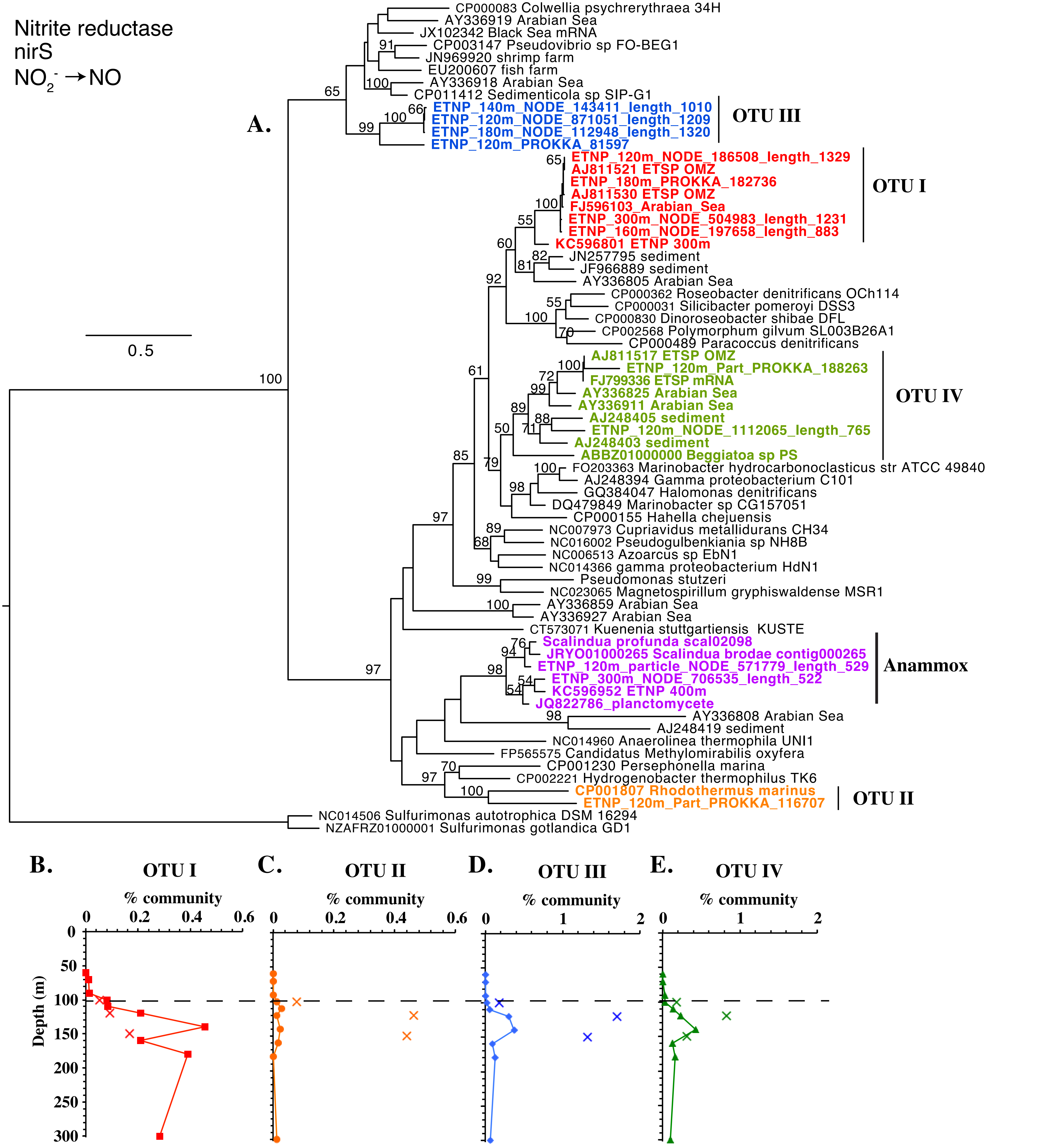

Figure S9. A) Phylogenetic tree of iron containing NO producing nitrite reductase *nirS*. Names with ETNP indicate sequences assembled from our metagenomes. Labels and colors on the tree match individual OTU depth profiles (B) OTU I, (C) OTU II, (D) OTU III, and (E) OTU IV. The depth profile for anammox bacteria is seen in Figure 1. In all depth profiles, Xs indicate particulate (>30 µm) samples. Dashed line indicates the top of the ODZ. % Community is calculated in comparison to the single copy core gene RNA polymerase (*rpoB*).
