# Supplementary material for: Niche Partitioning of the N Cycling Microbial Community of an Offshore Oxygen Deficient Zone
Source: Front Microbiol. 2017 Dec 5;8:2384. doi: 10.3389/fmicb.2017.02384 (PMC5723336; doi:10.3389/fmicb.2017.02384)
Supplement: Supplementary file 10 [file Image10.PDF]

Group	Subgroup	Organism	321	507	558	580	745
qnorB	A	Geobacillus stearothermophilus	MFGALLAHYYTEPD---SFFG	IHLWVEG	GHHYYY	LEVIP	PDT
		Staphylococcus aureus	LLGELLAHYYVENK----FFG	VHLWVEG	GHHYYW	LEVVP	WDI
	B	Herminiimonas arsenicoxydans	FLGGLTAHYTIEGQ---HFYG	VHLWVEG	FHHMYF	LEVVP	GDV
		Hahella chejuensis	LLGGFTAHYTVEGH---HFYG	VHLWVEG	FHHLYF	LEVVP	GDV
		Cellvibrio japonicus	LLGGFTAHYTIEGQ---QFYG	VHLWVEG	FHHLYF	LEVVP	GDV
		Polaromonas naphthalenivorans	GMGVITAHYAVEGQ---SFFG	VHLWVEG	LHHLYF	LEVVP	GDV
		Parvibaculum lavamentivorans	LLGAMTAHYQIEGQ---LVYG	VHLWVEG	LHHLYF	LEVVP	GDT
		Xanthobacter autotrophicus	LLGIVTAHYAVEGQ---GLYG	VHLWVEG	FHHLYF	LEVVP	GDV
nod		Methylomirabilis oxyfera p2434	LAGILSAEDFVGGGPGSAIAT	IHMWVEV	SHNFYW	MQVLP	GGA
		Methylomirabilis oxyfera p2437	LAGILGAEDFVGGGPGEAILG	IHMWVEV	SHNFYW	MQVLP	GGV
	I	ETNP 160m PROKKA 33601	IAGLLTVADFTHFF---AKYD	VHMWVEA	SHNFYW	LQVAP	GVV
		ETNP 120m free PROKKA 265821	IAGLLTVADFTHFF---AKYD	VHMWVEA	SHNFYW	LQVAP	GVV
	II	ETNP 120m PROKKA 89302	LAGIIIATDFVRPF------G	VHMWVEV	SHNFYW	LQVLP	GGL
		ETNP 120m Part PROKKA 50886	LGGVIAATDFVRPG------G	VHMWVEV	SHNFYW	LQVLP	GGA
	III	ETNP 300m PROKKA 194139	LAGIISATDFIRPF------G	VHMWVEV	SHNFYW	LQVLP	GGG
		ETNP 300m Part PROKKA 193327	LAGIIGASDFIRPF------G	VHMWVEV	SHNFYW	LQVLP	GGG
		ETNP 2013 Stn6 300m	LAGIIGASDFIRPF	VHMWVEV	SHNFYW	LQVLP	GGG
	IV	ETNP 120m PROKKA 15008	FAGIACSIDFVRPW------G	VHMWVEV	GHNFYW	TQVLP	GGH
qnorB-like		Algoriphagus mannitolivorans	LAGVLTVHDFVGFT---SFFG	VHMWVEA	SHNFYW	LQVVP	GGS
		Mariniradius saccharolyticus	LAGVLTVHDFVGFT---NFFG	VHMWVEA	SHNFYW	LQVVP	GGS
		Indibacter alkaliphilus	LAGVLTVHDFVGFT---KFFG	VHMWVEA	SHNFYW	LQVVP	GGA
		Cecembia lonarensis	LAGVLTVHDFVGFT---KFFG	VHMWVEA	SHNFYW	LQVVP	GGA
		Muricauda ruestringensis	SSGFVTINEFVDYL---GFFG	VHMWVEA	SHNFYW	LQFVP	GAC
		Arenibacter algicola	SSGFITINEFIDYL---GYFG	VHMWVEA	SHNFYW	LQFVP	GAC
		Chlorobi bacterium OLB7	LAGILTVHDFVGFV---NFFG	VHMWVEA	SHNFYW	LQVIP	GGA
		Bacteroidetes bacterium OLB12	LAGVLTVHDFVGFV---NFFG	VHMWVEA	SHNFYW	LQVVP	GGS
		Flavohumibacter sp ZG627	LAGILTVHDFVGFV---NFFG	VHMWVEA	SHNFYW	LQVIP	GGS
		Sediminibacterium sp OR53	LAGILTVHDFVGFV---HFFG	VHMWVEA	SHNFYW	LQVIP	GGA

Figure S10. Alignment showing the active site (red) and quinol binding site (blue) for qnorB, putative nod, qnorB-like, and assembled ETNP sequences. Residues comprising the active site were determined by modeling in Ettwig et al (2012). Colored boxes indicate key residues indicated in Ettwig et al (2012). Numbers delineate the residue number in *G. stearothermophilus*. Accession numbers are found on the phylogenetic tree in Figure 7. The active and binding sites for ETNP 2013 Stn6 300m is found in Padilla et al. (2016).
