# Supplementary material for: Niche Partitioning of the N Cycling Microbial Community of an Offshore Oxygen Deficient Zone
Source: Front Microbiol. 2017 Dec 5;8:2384. doi: 10.3389/fmicb.2017.02384 (PMC5723336; doi:10.3389/fmicb.2017.02384)
Supplement: Supplementary file 11 [file Image11.PDF]

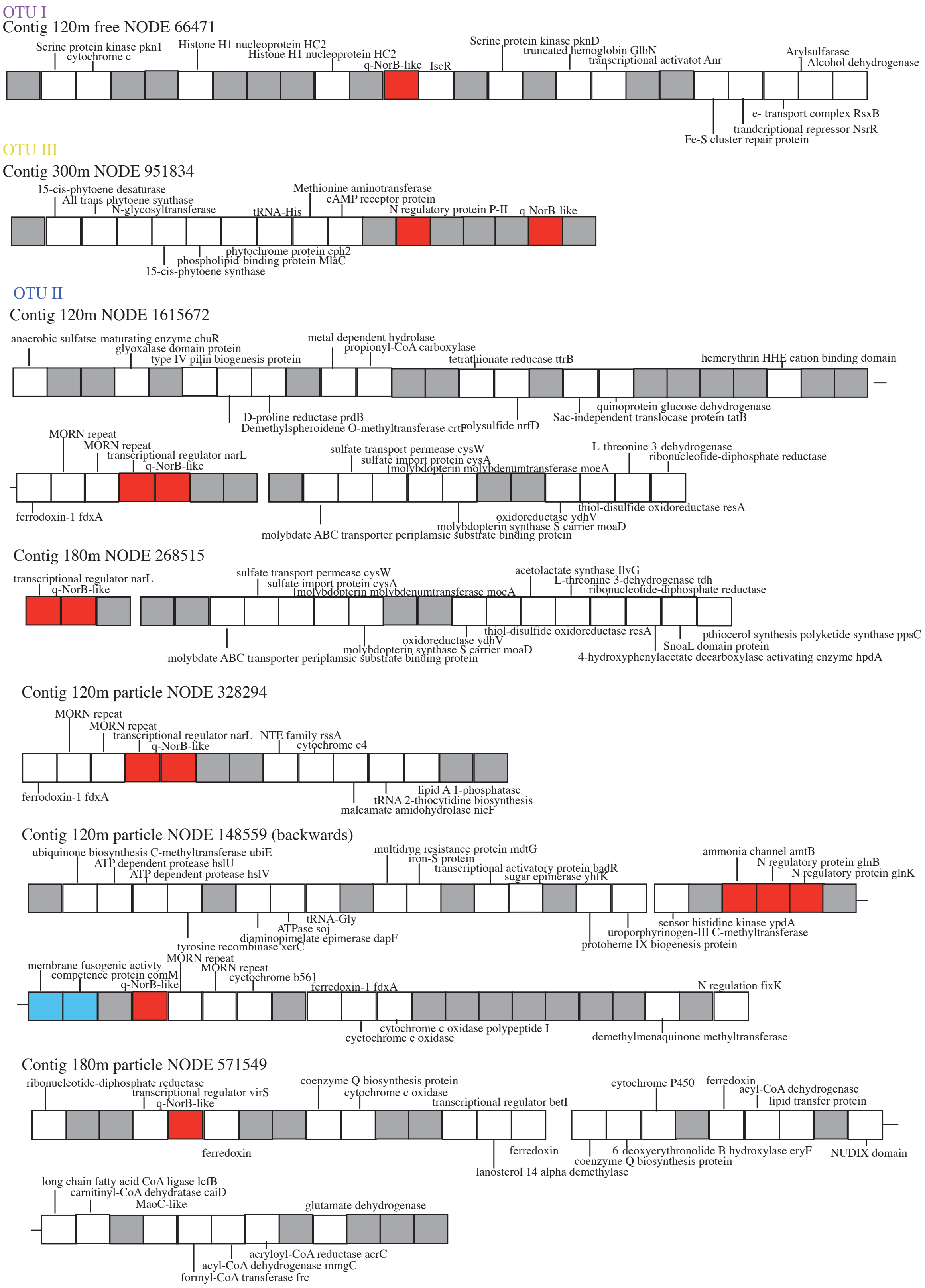

Figure S11. Schematic of selected contigs containing *q-norB*-like genes. Hypothetical proteins are shown in gray. Proteins related to N cycling are shown in red. Contigs are clustered by the phylogenetic affiliation of their *q-norB* gene.
