# Supplementary material for: Niche Partitioning of the N Cycling Microbial Community of an Offshore Oxygen Deficient Zone
Source: Front Microbiol. 2017 Dec 5;8:2384. doi: 10.3389/fmicb.2017.02384 (PMC5723336; doi:10.3389/fmicb.2017.02384)
Supplement: Supplementary file 12 [file Image12.PDF]

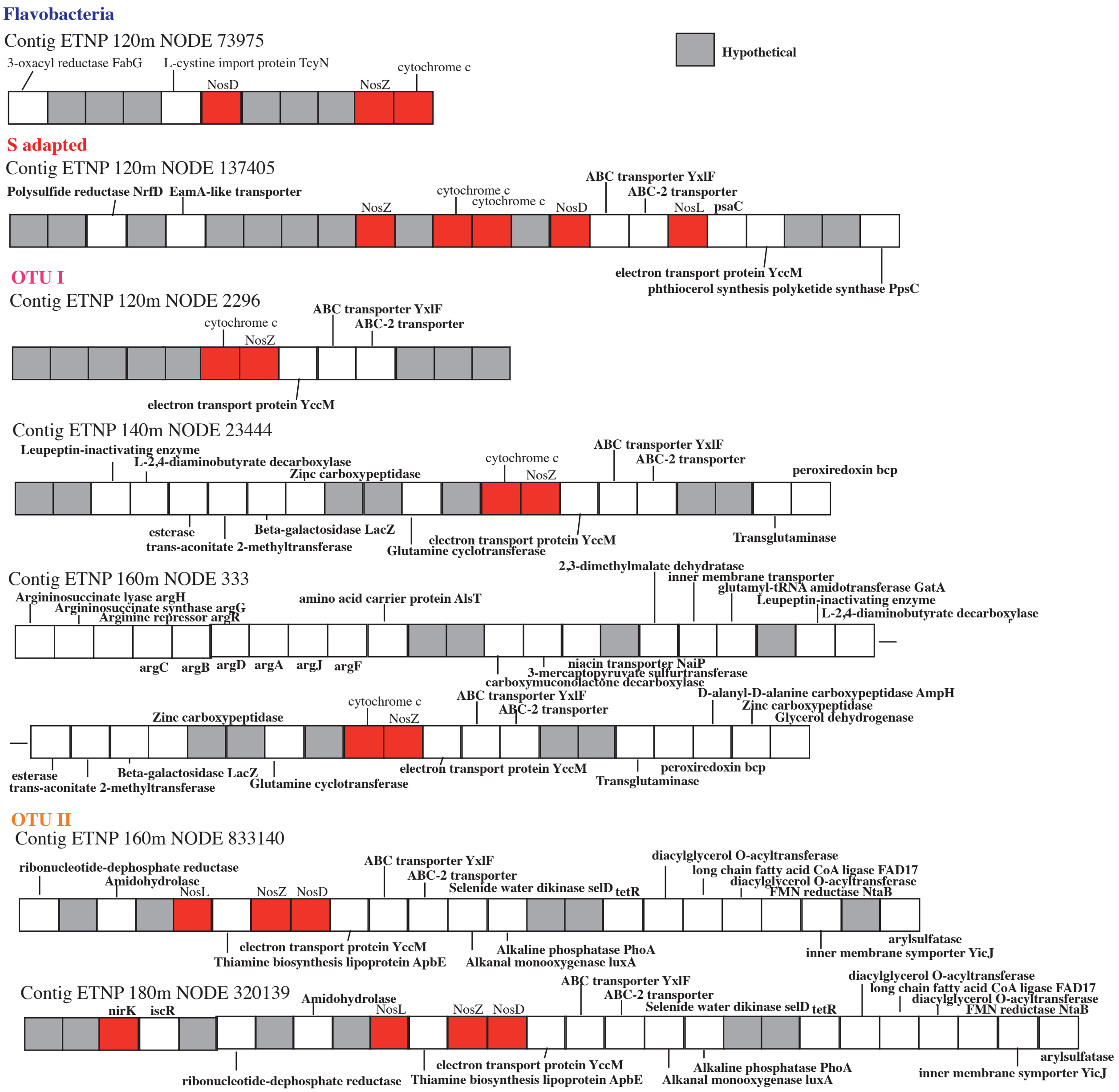

Figure S12. Schematic of selected contigs containing *nosZ* gene. Hypothetical proteins are shown in gray. Proteins related to N_2O reduction are shown in red. Contigs are clustered by their phylogenetic affiliation.
