# Supplementary material for: Niche Partitioning of the N Cycling Microbial Community of an Offshore Oxygen Deficient Zone
Source: Front Microbiol. 2017 Dec 5;8:2384. doi: 10.3389/fmicb.2017.02384 (PMC5723336; doi:10.3389/fmicb.2017.02384)
Supplement: Supplementary file 13 [file Image13.PDF]

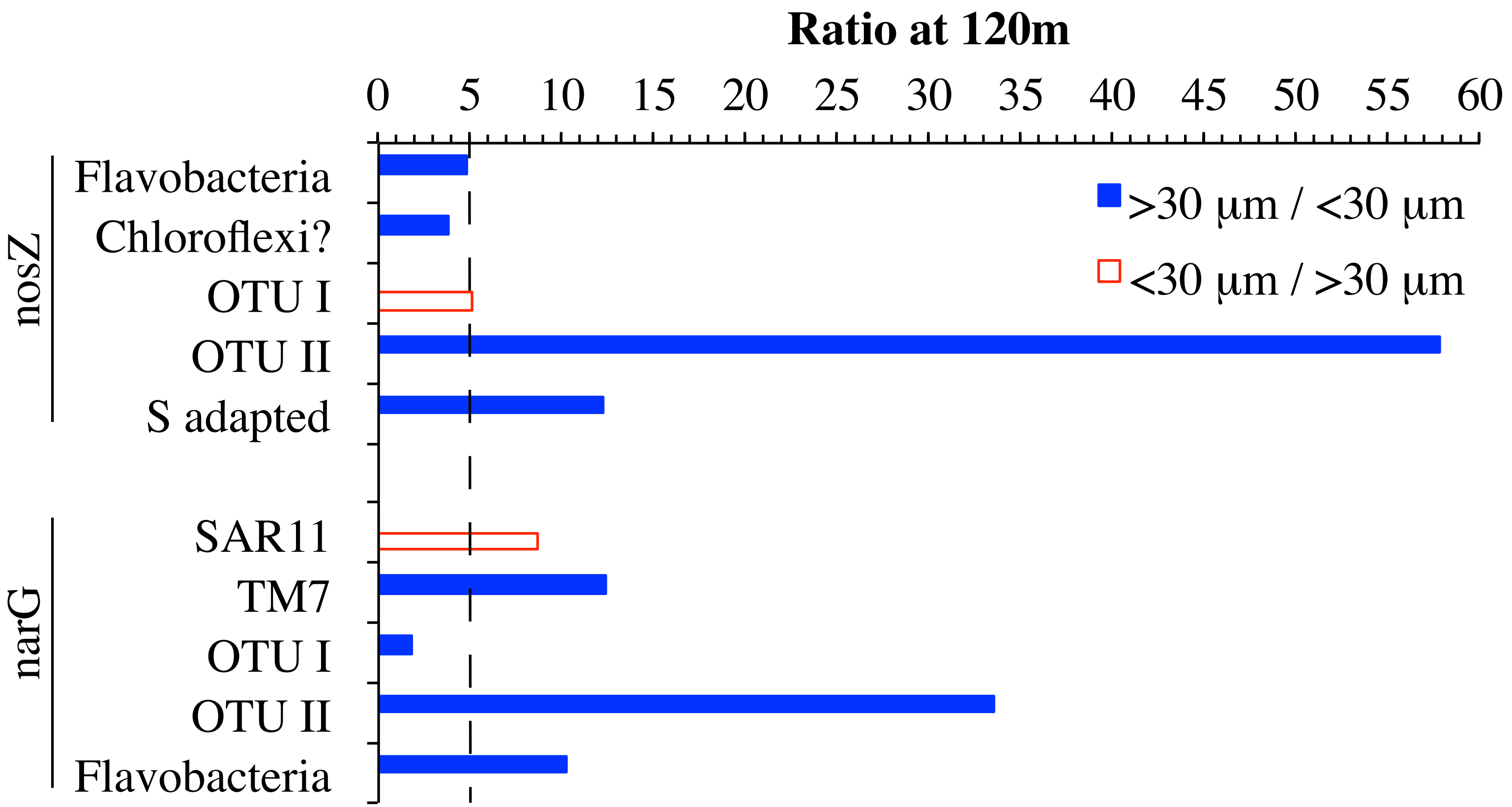

Figure S13. nosZ and narG phylotypes on particle and prefiltered samples from 120m. If the ratio of >30 µm / <30 µm is >5, then the phytotype is considered particle-attached, and if the ratio of <30 µm / >30 µm is >5, then the phylotype is considered free-living (Fuchsman et al., 2011).
