# Supplementary material for: Niche Partitioning of the N Cycling Microbial Community of an Offshore Oxygen Deficient Zone
Source: Front Microbiol. 2017 Dec 5;8:2384. doi: 10.3389/fmicb.2017.02384 (PMC5723336; doi:10.3389/fmicb.2017.02384)
Supplement: Supplementary file 14 [file Image14.PDF]

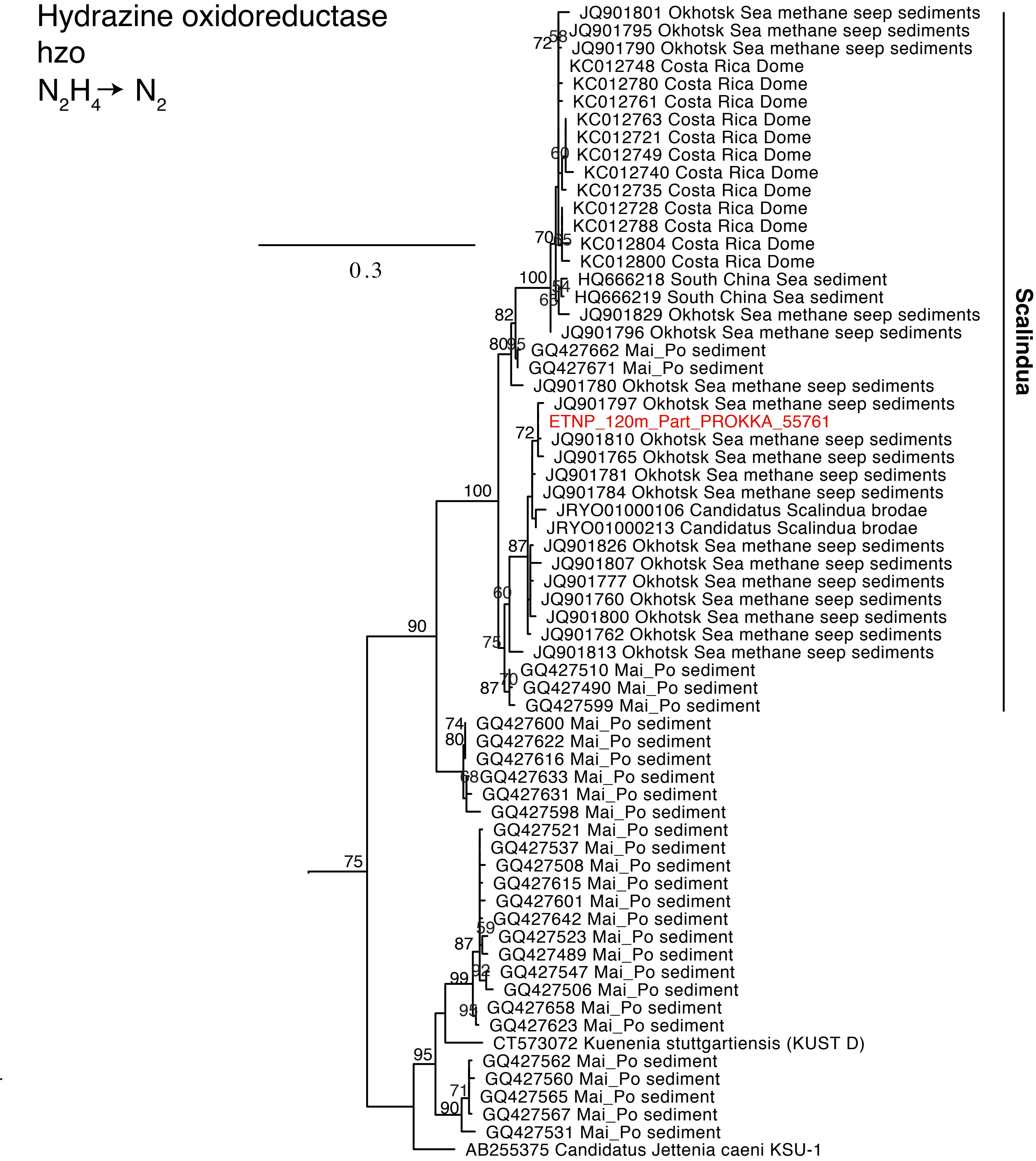

Figure S14. Phylogenetic tree of the anammox hydrazine oxidoreductase (*hzo)* gene. Names with ETNP indicate sequences assembled from our metagenomes. Only one phylotype was found for this gene. Outgroup (not shown) is *hao*.
