# Supplementary material for: Niche Partitioning of the N Cycling Microbial Community of an Offshore Oxygen Deficient Zone
Source: Front Microbiol. 2017 Dec 5;8:2384. doi: 10.3389/fmicb.2017.02384 (PMC5723336; doi:10.3389/fmicb.2017.02384)
Supplement: Supplementary file 15 [file Table1.DOCX]

Table S1. Information about metagenomic sequence data. Particle data is from >30 µm filter. Free-living is <30 µm. Other samples are whole water.

| Sample | St | Read length | # read pairs | % post QC | Assembly N50 (bp) | % Reads assembled  /Mb | rpoB reads |
| --- | --- | --- | --- | --- | --- | --- | --- |
| 60m | 136 | 150 | 68,855,288 | 67.94 | 836 | 6%/28 | 17412 |
| 70m | 136 | 125 | 60,315,861 | 98.62 | 1074 | 14%/113 | 20235 |
| 90m | 136 | 125 | 52,708,497 | 98.29 | 1344 | 17%/173 | 16922 |
| 100m | 136 | 150 | 27,328,776 | 91.27 | 1301 | 24%/85 | 19747 |
| 110m | 136 | 150 | 16,791,330 | 86.76 | 1348 | 24%/45 | 8076 |
| 120m | 136 | 125 | 74,367,303 | 99.00 | 1705 | 35%/298 | 70214 |
| 140m | 136 | 150 | 34,561,114 | 93.01 | 1389 | 32%/135 | 18881 |
| 160m | 136 | 125 | 64,618,906 | 98.76 | 1841 | 32%/204 | 78521 |
| 180m | 136 | 125 | 54,217,191 | 98.73 | 1886 | 34%/214 | 57209 |
| 300m | 136 | 125 | 63,470,107 | 98.87 | 1703 | 33%/169 | 86979 |
| 120m_free | BB2 | 125 | 158,967,556 | 98.92 | 1617 | 35%/292 | 180588 |
| 120m_particle | BB2 | 125 | 128,725,770 | 98.62 | 1245 | 15%/223 | 12249 |
| 100m_particle | BB2 | 125 | 95,866,006 | 98.32 | 1301 | 11%/131 | 8740 |
| 150m_particle | BB2 | 125 | 105,643,472 | 98.78 | 895 | 8%/41 | 10784 |
