# Supplementary material for: Niche Partitioning of the N Cycling Microbial Community of an Offshore Oxygen Deficient Zone
Source: Front Microbiol. 2017 Dec 5;8:2384. doi: 10.3389/fmicb.2017.02384 (PMC5723336; doi:10.3389/fmicb.2017.02384)
Supplement: Supplementary file 16 [file Table2.DOCX]

Table S2. Type of published data and references used for comparison in this paper.

| **Type of Data** | **Reference** | **Notes** |
| --- | --- | --- |
| N_2_O concentrations | Peng et al., 2015; Babbin et al., 2015 | St BB2, 2012 |
| Ammonium and nitrite oxidation rates | Peng et al., 2015 | St BB2, 2012 |
| qPCR of *amoA* | Peng et al., 2015 | St BB2, 2012 |
| Ladderane Lipids | Sollai et al., 2015 | St 136, 2012 |
| N_2_O reduction rates | Babbin et al., 2015 | St BB2, 2012 |
| Total N_2_ production rates | Babbin et al., 2014 | St BB2, 2012 |
| Nitrite and CTD oxygen concentrations | Babbin et al., 2015 supplemental | Entire transect, 2012 |
| STOX oxygen concentrations | Tiano et al., 2014 | St BB2, 2012 |
| Transcript sequences | Ganesh et al., 2015 | Data from a 2013 ETNP cruise re-analyzed here. |
