# Supplementary material for: Niche Partitioning of the N Cycling Microbial Community of an Offshore Oxygen Deficient Zone
Source: Front Microbiol. 2017 Dec 5;8:2384. doi: 10.3389/fmicb.2017.02384 (PMC5723336; doi:10.3389/fmicb.2017.02384)
Supplement: Supplementary file 3 [file Image3.PDF]

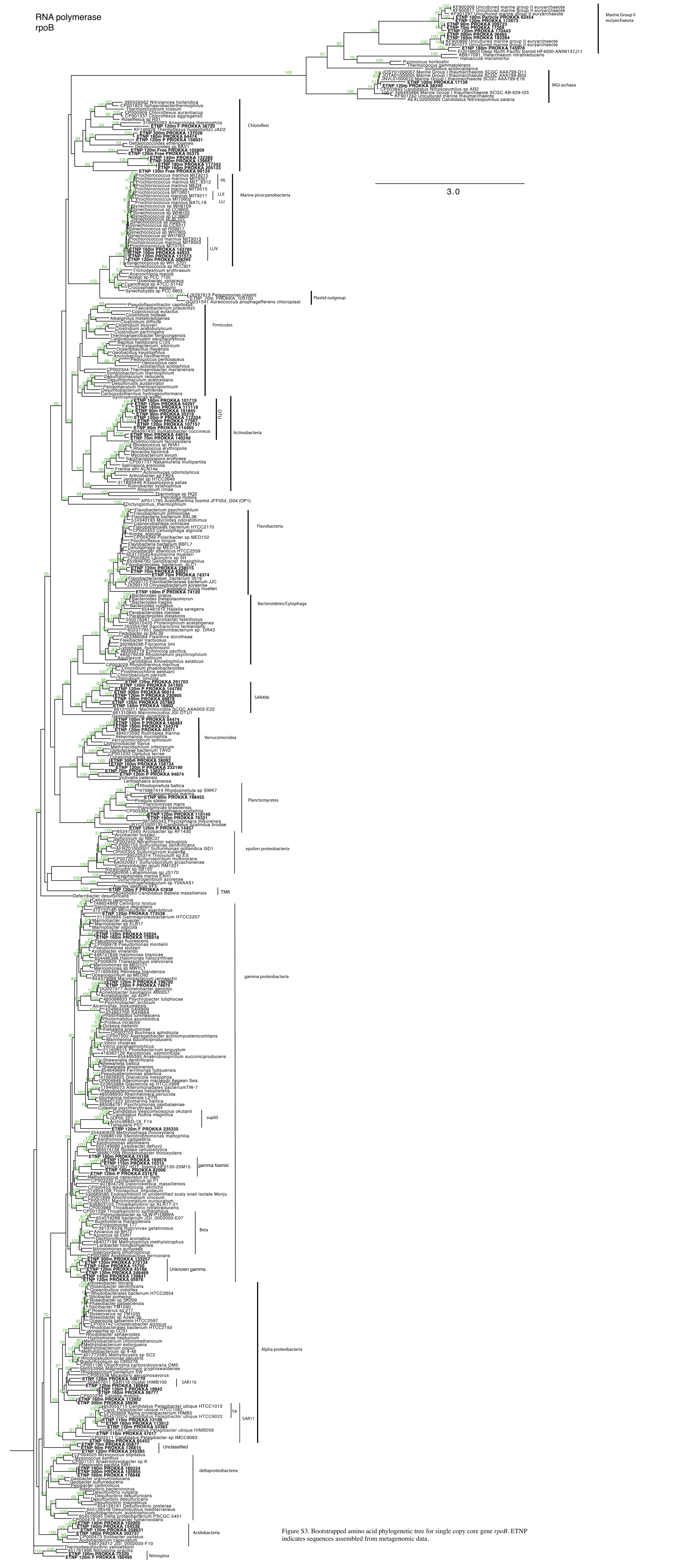

Figure S3. Bootstrapped amino acid phylogenetic tree for single copy core gene *rpoB*. ETNP indicates sequences assembled from metagenomic data.
